# Mechanochemical Synthesis of Primary Amides

**DOI:** 10.1021/acs.joc.1c02350

**Published:** 2021-10-01

**Authors:** Jorge Gómez-Carpintero, J. Domingo Sánchez, J. Francisco González, J. Carlos Menéndez

**Affiliations:** Unidad de Química Orgánica y Farmacéutica, Departamento de Química en Ciencias Farmacéuticas, Facultad de Farmacia, Universidad Complutense, 28040 Madrid, Spain

## Abstract

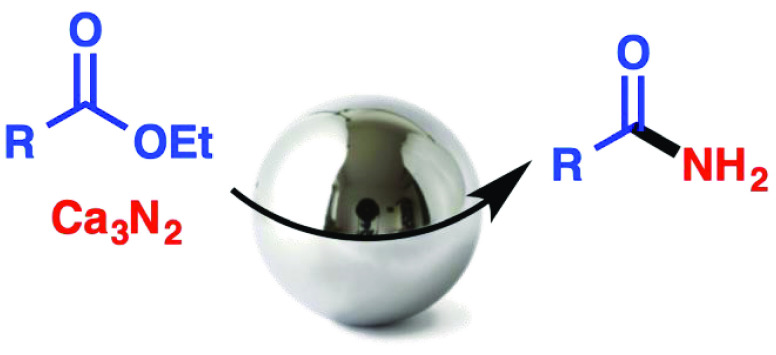

Ball milling of aromatic, heteroaromatic, vinylic, and aliphatic esters with ethanol and calcium nitride afforded the corresponding primary amides in a transformation that was compatible with a variety of functional groups and maintained the integrity of a stereocenter α to carbonyl. This methodology was applied to α-amino esters and *N*-BOC dipeptide esters and also to the synthesis of rufinamide, an antiepileptic drug.

Mechanochemistry involves chemical transformations induced by the direct absorption of mechanical energy, normally arising from grinding or milling processes. This mode of activation has grown in importance over the past decade,^1^ and indeed, in 2019 IUPAC chose mechanochemistry as one of the “ten chemical innovations that will change the world”.^2^ Mechanochemistry is generally performed in the solid state and, therefore, under solvent-free conditions with very high reagent concentrations. Consequently, solvation phenomena are not significant, often leading to accelerated reactions and alterations in product selectivity, which may lead to the discovery of new chemical transformations.^3^ Furthermore, the use of solvent-free conditions is relevant in the context of green chemistry, since volatile organic solvents are the vast majority of residues from synthetic activities.^4^ Accordingly, there is a growing interest in the application of mechanochemistry to the synthesis of active pharmaceutical ingredients (APIs).^5^

The amide group is one of the most fundamental structural fragments in organic molecules. It is key to the primary structure of peptides and proteins, a group of biomolecules essential to life, and it is also widespread in polymers, agrochemicals, and drug molecules. Indeed, amide preparation is the most frequent chemical transformation in drug synthesis, and it has been estimated to comprise about 25% of the reactions performed in medicinal chemistry projects.^6^ A number of mechanochemical approaches to amide synthesis from amines and carboxylic acids have been developed on the basis of the use of coupling reagents such as EDC,^7^ EDC/HOBt in the presence of Mg–Al hydrotalcite,^8^ EDC in the presence of nanocrystalline hydroxyapatite,^9^ carbonyldiimidazole,^10^ 2,4,6-trichloro-1,3,5-triazine/triphenylphosphine,^11^ uronium-based reagents,^12^ and hydrolytic enzymes.^13^ In polymer science, amide bonds have also been generated from acyl chlorides and anilines under mortar and pestle milling conditions^14^ or ball milling.^15^*N*-Arylamides have been obtained from *O*-pivaloyl hydroxamic acids and aryl iodides or boronic acids under ball milling in the presence of a copper mediator.^16^ Mechanochemical versions of the Ritter reaction^17^ and the Beckmann rearrangement^18^ are also relevant routes to amides. For the case of peptide synthesis, direct coupling of amino acids with Leuchs anhydrides or *N*-carboxyanhydrides,^19^ or the use of ethyl cyano(hydroxyimino)acetate (Oxyma) to promote the reaction between amino ester salts and *N*-protected amino acids, have been employed.^20^ Nevertheless, the synthesis of primary amides by mechanochemical methods has not been described so far, with the exception of the transformation of carboxylic acids into amides by manually grinding carbocylic acids, 2,4,6-trichloro-1,3,5-triazine, and ammonium thiocyanate in the presence of potassium carbonate,^21^ perhaps due to the difficulties found in handling gaseous reagents under ball milling.^22^ Our goal here is to offer a simple solution to this problem via a process for preparing primary amides from esters using calcium nitride as a source of ammonia. Primary amide groups are widespread in natural products and drug molecules, and some representative examples of the latter are shown in Figure 1.

**Figure 1:**
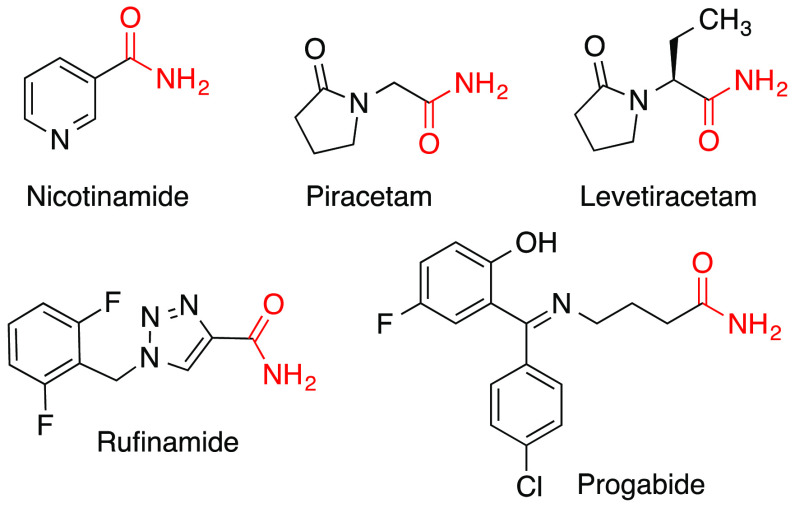
Representative drugs containing a primary amide group.

We first optimized the mechanochemical transformation of ethyl 3-fluorobenzoate **1a** into primary amide **2a** with magnesium nitride, which has been employed as a source of ammonia in several transformations, although harsh reaction conditions were required (sealed tube, 80 °C, 24 h).^23^ We first examined the reaction of **2a** with magnesium nitride–ethanol in the absence of additives, but only starting materials were recovered at milling frequencies up to 30 Hz for 90 min using a stainless steel milling jar and ball (Table 1, entry 1). After several Lewis acid catalysts were screened with moderate success (entries 2–5), zinc chloride (0.2 equiv, 90 min, 30 Hz) gave a good 85% conversion (entry 6), although attempts to reduce reaction time, catalyst loading, or milling frequency were unsuccessful (entries 7–9). Indium trichloride was later identified as a better catalyst, allowing 91% conversion (entry 10), but again reductions in reaction time or milling frequency were slightly detrimental (entries 11 and 12) and a lower amount of catalyst was not tolerated (entry 13).

**Table 1:**
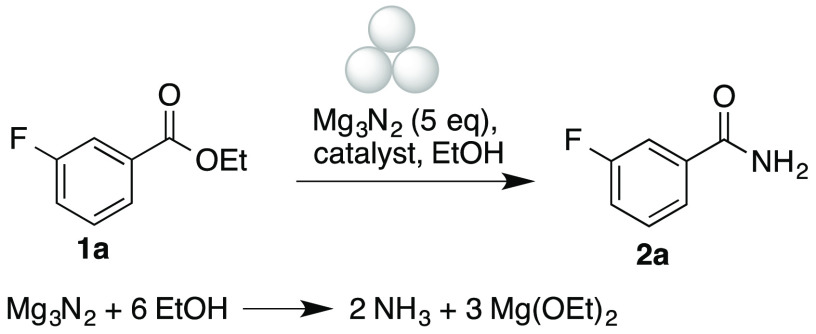
Catalyst Optimization in the Synthesis of **2a**

entry	catalyst (equiv)	time (min)	frequency^a^ (Hz)	conversion^b^^,^^c^ (%)
1		90	30	0
2	AlCl_3_ (0.2)	90	30	37
3	Yb(OTf)_3_ (0.2)	90	30	28
4	CuSO_4_ (0.2)	90	30	12
5	CuBr (0.2)	90	30	67
6	ZnCl_2_ (0.2)	90	30	85
7	ZnCl_2_ (0.2)	60	30	52
8	ZnCl_2_ (0.1)	90	30	43
9	ZnCl_2_ (0.1)	90	25	37
**10**	**InCl**_**3**_**(0.2)**	**90**	**30**	**91**
11	InCl_3_ (0.2)	60	30	86
12	InCl_3_ (0.2)	60	25	83
13	InCl_3_ (0.1)	60	25	23

a In a 10 mL stainless steel milling jar containing a single stainless steel ball 10 mm in diameter.

b Estimated by ^1^H NMR analysis of the reaction crude, using 1,3,5-trimetoxybenzene as an internal standard.

c 5 equiv of magnesium nitride and 1 mL of ethanol were employed.^23a^

Starting from these conditions, the optimization of other reaction parameters was undertaken. Attempted purification of the crude reaction products by extraction with ethyl acetate–water, in spite of the excellent conversion, gave only 22% isolated yield (Table 2, entry 1). This was ascribed to interference of magnesium salts present in the reaction medium, which remain as a suspension during workup. We attempted the use of alternative sources of ammonia, such as ammonium chloride or acetate, but no product formation was observed (entries 2 and 3). Lowering the amount of magnesium nitride to 2 equiv was also unsuccessful (entries 4 and 5), but the use of 3 equiv of the ammonia source, coupled to an increase in the catalyst load, gave a promising 73% isolated yield (entry 6). In an effort to improve the isolation protocol, we performed workup with an aqueous solution of Rochelle salt, which often prevents the formation of emulsions in reactions involving aluminum-based reagents, but in our case, no improvement was observed. A control experiment carried out in solution, at room temperature, gave 49% isolated yield of compound **2a**, proving the beneficial effect of the mechanochemical conditions (entry 7). The use of calcium nitride was then assayed, with improved results (entries 8 and 9). Finally, we observed an additional improvement by lowering the amount of ethanol (entries 10 and 11), which can be ascribed to more efficient milling due to the smaller amount of liquid in the mixture.

**Table 2:** Optimization of Additional Reaction Parameters in the Synthesis of **2a**^a^

entry	catalyst (equiv)	ammonia source (equiv)	proton source	yield (%)
1	InCl_3_ (0.2)	Mg_3_N_2_ (5)	EtOH (1 mL)	23
2	InCl_3_ (0.2)	NH_4_Cl (5)		0
3	InCl_3_ (0.2)	NH_4_OAc (5)		0
4	InCl_3_ (0.2)	Mg_3_N_2_ (2)	EtOH (1 mL)	traces
5	InCl_3_ (0.3)	Mg_3_N_2_ (2)	EtOH (1 mL)	traces
6	InCl_3_ (0.4)	Mg_3_N_2_ (3)	EtOH (1 mL)	73
7	InCl_3_ (0.4)	Mg_3_N_2_ (3)	EtOH (solvent)	49^b^
8	InCl_3_ (0.4)	Ca_3_N_2_ (2)	EtOH (1 mL)	10
9	InCl_3_ (0.4)	Ca_3_N_2_ (3)	EtOH (1 mL)	79
10	InCl_3_ (0.4)	Ca_3_N_2_ (3)	EtOH (0.5 mL)	83
11	**InCl**_**3**_**(0.4)**	**Ca**_**3**_**N**_**2**_**(3)**	**EtOH(0.3 mL)**	**88**

a All reactions were performed at 1 mmol scale by ball milling (stainless steel jar and ball) at 30 Hz for 90 min.

b Control experiment carried out in solution.

Using the optimized conditions, we examined the scope of the mechanochemical protocol for the synthesis of primary amides. Our results are summarized in Scheme 2a and show that the method is suitable for the preparation of aromatic (**2a**–**c**), heteroaromatic (**2h**–**j**), vinylic (**2d**), aliphatic (**2e**,**f** and **2k**–**n**), and aliphatic cyclic (**2g**) amides from the corresponding esters. Several functional groups, including halogen, acetal, thioether, and amino groups, were tolerated (**2a**, **2e**, and **2m**,**n**, respectively). It is also relevant to note that the stereogenic center of the amino acid esters maintained its integrity, as shown by the optical rotation of compound **2m**, identical to published values. The mechanochemical conditions were also employed to obtain dipeptide amides from the corresponding esters while maintaining the carbamate protection, affording compounds **2o**–**2q**. A lactone, 3,4-dihydrocoumarin **1q**, was also efficiently transformed into the corresponding hydroxy amide **2q** (Scheme 2b). A control experiment with benzoic acid did not furnish **2b**, giving instead recovered starting material.

**Scheme 2:**
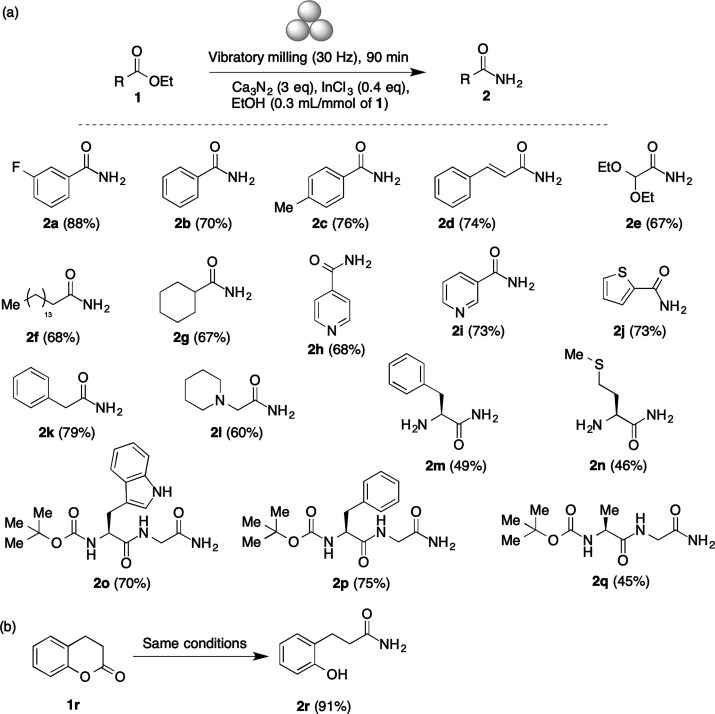
Scope of the Mechanochemical Primary Amide Synthesis

Yields were generally in the 70–90% range and could be compared to those obtained under sealed-tube heating^23a^ for three examples. In the case of **2d** and **2g**, the mechanochemical yield was lower (74% vs 99% for **2d** and 67% vs 85% for **2g**), while **2n** gave a better yield under ball milling (91% vs. 83%). Importantly, the mechanochemical conditions were milder (no external heating vs 80 °C), reaction times were drastically shorter (90 min vs 24 h), and solid-phase extraction through a hydrophobic frit^23a^ was not required for purification.

In order to show the applicability of the mechanochemical method to a multistep synthesis, we examined the preparation of the drug rufinamide,^24^ approved for the treatment of Lennox–Gastaut syndrome and other forms of epilepsy, by the route summarized in Scheme 3. The starting material **3** was transformed into azide **4** and then into the triazole-derived ester **5** using a literature procedure,^25^ and its mechanochemical treatment with calcium nitride under our optimal conditions afforded rufinamide (**6**) in 61% yield.

**Scheme 3:**
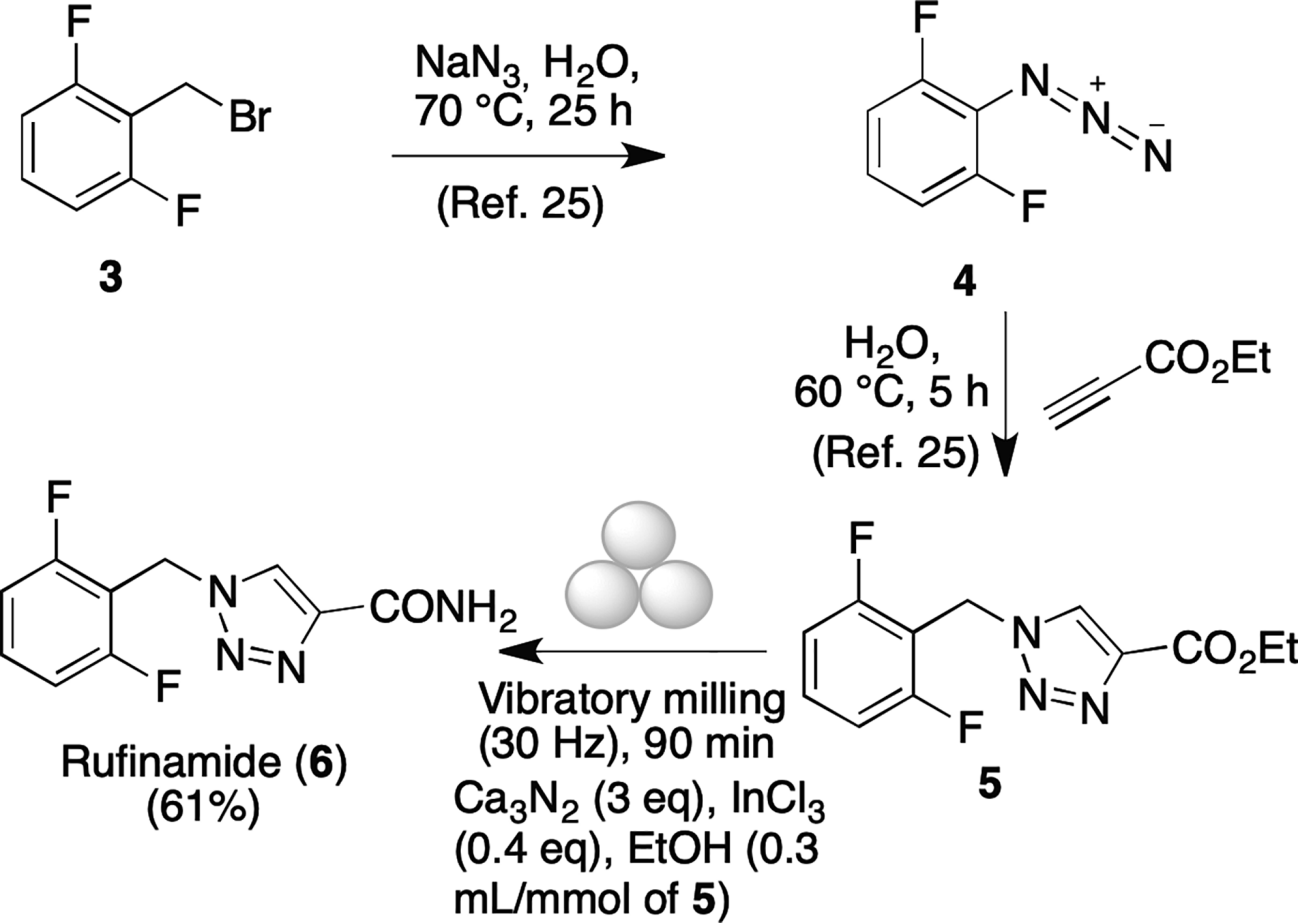
Synthesis of the Antiepileptic Drug Rufinamide Using Mechanochemical Conditions for the Primary Amide Formation Step

In conclusion, mechanochemical activation by mall milling is a suitable method for the transformation of esters into primary amides by reaction with calcium nitride that does not require chromatography. It is compatible with a variety of functional groups and a stereocenter adjacent to carbonyl and was applied to the synthesis of *N*-BOC dipeptide esters and the antiepileptic drug rufinamide.

## Experimental Section

*Caution! The use of magnesium nitride to transform esters into amides in methanol solution at 80 °C has been described to cause occasional explosions, especially when working at a relatively large scale involving the use of 1.3 g of magnesium nitride and 6 mL of methanol; see the discussion in ref (37). We thank Duncan Browne (UCL School of Pharmacy) for bringing to our attention this safety issue and the associated references. Under our conditions (calcium nitride below 400 mg, ethanol, ball milling in screw-top grinding jars) we have never observed any explosion.*

### General Experimental Information

All reagents and solvents were of commercial quality and were used as received. All compounds **1** were of commercial origin, with the exception of **1o**–**q**, which were prepared using a literature method.^26^ Reactions were monitored by thin-layer chromatography on aluminum plates coated with silica gel and a fluorescent indicator. Mechanochemical reactions were performed in a mixer mill at a 30 Hz frequency, using a 10 mL stainless steel grinding jar and a single stainless steel ball 15 mm in diameter. Infrared spectra were recorded with a ATR spectrophotometer equipped with a diamond accessory. NMR spectra were recorded using spectrometers operating at 250/300 MHz for ^1^H NMR and 62.5/75 MHz for ^13^C NMR, maintained by the Nuclear Magnetic Resonance Unit at Universidad Complutense (UCM). Optical rotation values were determined with a polarimeter having a 1 mL cell and a sodium vapor lamp. Combustion elemental analyses were determined by the Elemental Microanalysis Unit (UCM).

### General Procedure for the Mechanochemical Synthesis of Primary Amides

The suitable ester (1 mmol), together with Ca_3_N_2_ (445 mg, 3 mmol), InCl_3_ (88 mg, 0.4 mmol), and ethanol (0.3 mL), were added to a 10 mL stainless steel milling jar, along with a 10 mm stainless steel ball. The ball mill was set to vibrate at a frequency of 30 Hz for 90 min. Subsequently, the contents of the milling jar were washed twice with ethyl acetate (5 mL) and water (2 mL), and the two layers were separated. The aqueous layer was extracted with ethyl acetate (2 × 5 mL), and the combined organic fractions were dried with Na_2_SO_4_ and concentrated under vacuum. The resulting solid was washed with petroleum ether (2 × 2.5 mL) to afford the pure amides. The compounds thus obtained, including **2a**–**2c**,^27^**2d**, **2g** and **2r**,^23a^**2e**,^28^**2f**,^29^**2h** and **2j**,^30^**2i**,^31^**2k**,^32^**2l**,^33^ and **6**(34) showed characterization data coincident with those found in the literature, and copies of their NMR spectra can be found in the Supporting Information.

(+)-Phenylalaninamide (**2m**). ^1^H NMR (300 MHz, methanol-*d*_4_): δ 7.35–7.19 (m, 5H), 3.63–3.53 (m, 1H), 3.04 (dd, *J* = 13.4, 5.9 Hz, 1H), 2.81 (dd, *J* = 13.4, 7.6 Hz, 1H). ^13^C{^1^H} NMR (75 MHz, methanol-*d*_4_): δ 178.1, 137.5, 129.1, 128.2, 126.4, 56.0, 41.1. [α]_D_^25^ = +22.4 (0.02 g/100 mL, H_2_O + 3 drops of 35% HCl) [lit.^35^ = +20.0 (for the hydrochloride salt, 2 g/100 mL, H_2_O)].

(+)-Methionamide **(2n)**. Spectral data were coincident with those found in the literature.^36^ [α]_D_^25^ (0.005 g/100 mL, H_2_O + 3 drops of 35% HCl) = +6.6.

(*S*)-*tert*-Butyl (1-((2-Amino-2-oxoethyl)amino)-3-(1*H*-indol-3-yl)-1-oxopropan-2-yl)carbamate (**2o**). IR: 3296, 3066, 2976, 1655 cm^–1^. ^1^H NMR (250 MHz, CDCl_3_) δ 9.00 (br s, 1H), 7.57 (d, *J* = 7.7 Hz, 1H), 7.31 (d, *J* = 8.6 Hz, 1H), 7.19–7.03 (m, 3H), 6.99 (s, 1H), 6.42 (br s, 1H), 6.25 (br s, 1H), 5.58 (br s, 1H), 4.44 (q, *J* = 6.5 Hz, 1H), 3.62 (br s, 2H), 1.39 (s, 9H). ^13^C{^1^H} NMR (62.5 MHz, CDCl_3_) δ 173.4, 172.8, 156.4, 136.7, 127.7, 124.1, 122.5, 119.94, 118.9, 112.0, 110.1, 81.0, 56.1, 43.0, 28.7 ppm. [α]_D_^25^ = +6.16 (*c* = 0.006 g/100 mL, CHCl_3_). Anal. Calcd for C_18_H_24_N_4_O_4_, C, 59.99; H, 6.71; N, 15.55. Found: C, 59.72; H, 6.59; N, 15.31.

(*S*)-*tert*-Butyl (1-((2-Amino-2-oxoethyl)amino)-1-oxo-3-phenylpropan-2-yl)carbamate (**2p**). IR: 3298, 3072, 2977, 1664 cm^–1^. ^1^H NMR (250 MHz, CDCl_3_, major conformer) δ 7.36–7.21 (m, 5H), 7.07 (br s, 1H, NH), 6.53 (br s, 1H), 5.90 (br s, 1H), 5.31 (br s, 1H), 4.37 (q, 1H, *J* = 7.0 Hz), 3.88 (m, 2H), 3.08 (m, 2H), 1.37 (s, 9H) ppm. ^13^C{^1^H} NMR (62.5 MHz, CDCl_3_) δ 172.6, 1721, 156.3, 136.8, 129.6, 129.2, 127.5, 81.1, 56.7, 43.1, 38.4, 28.6 ppm. [α]_D_^25^ = +1.37 (c = 0.006 g/100 mL, CHCl_3_). Anal. Calcd for C_16_H_23_N_3_O_4_, C, 59.80; H, 7.21; N, 13.08. Found: C, 59.45; H, 7.02; N, 12.79.

(*S*)-*tert*-Butyl (1-((2-Amino-2-oxoethyl)amino)-1-oxopropan-2-yl)carbamate (**2q**). IR: 3297, 2977, 1656 cm^–1^. ^1^H NMR (250 MHz, CDCl_3_ major conformer) δ 7.48 (br t, *J* = 5.0 Hz, 1H), 6.95 (br s, 1H), 6.26 (br s, 1H), 5.55 (br d, *J* = 5.0 Hz, 1H), 4.18 (m, 1H), 4.00 (dd, 2H, *J* = 17.0 and 5.5 Hz), 1.44 (s, 9H), 1.38 (d, 3H, *J* = 7.0 Hz) ppm. ^13^C{^1^H} NMR (62.5 MHz, CDCl_3_) δ 174.2, 172.6, 156.4, 80.9, 51.1, 43.1, 28.7, and 18.3 ppm. [α]_D_^25^ = −11.25 (*c* = 0.004 g/100 mL, CHCl_3_). Anal. Calcd for C_10_H_19_N_3_O_4_: C, 48.97; H, 7.81; N, 17.13. Found: C, 48.68; H, 7.62; N, 16.97.

## Supporting Information Available

Copies of ^1^H NMR and ^13^C{^1^H} NMR spectra of all compounds (PDF)
