# Supplementary material for: Mechanochemical Synthesis of Primary Amides
Source: J Org Chem. 2021 Oct 1;86(20):14232–7. doi: 10.1021/acs.joc.1c02350 (PMC8524419; doi:10.1021/acs.joc.1c02350)

Supporting information

Mechanochemical synthesis of primary amides

Jorge Gómez-Carpintero, J. Domingo Sánchez, J. Francisco González,* J. Carlos Menéndez*

Unidad de Química Orgánica y Farmacéutica, Departamento de Química en Ciencias Farmacéuticas, Facultad de Farmacia, Universidad Complutense, 28040 Madrid, Spain

Contents

Copies of spectra of compound **2a** S2

Copies of spectra of compound **2b** S3

Copies of spectra of compound **2c** S4

Copies of spectra of compound **2d** S5

Copies of spectra of compound **2e** S6

Copies of spectra of compound **2f** S7

Copies of spectra of compound **2g** S8

Copies of spectra of compound **2h** S9

Copies of spectra of compound **2i** S10

Copies of spectra of compound **2j** S11

Copies of spectra of compound **2k** S12

Copies of spectra of compound **2l** S13

Copies of spectra of compound **2m** S14

Copies of spectra of compound **2n** S15

Copies of spectra of compound **2o** S16

Copies of spectra of compound **2p** S17

Copies of spectra of compound **2q** S18

Copies of spectra of compound **2q** S19

Copies of spectra of compound **6** S20

3-Fluorobenzamide (2a)

^{1}H NMR (250 MHz, DMSO-d$_6$)

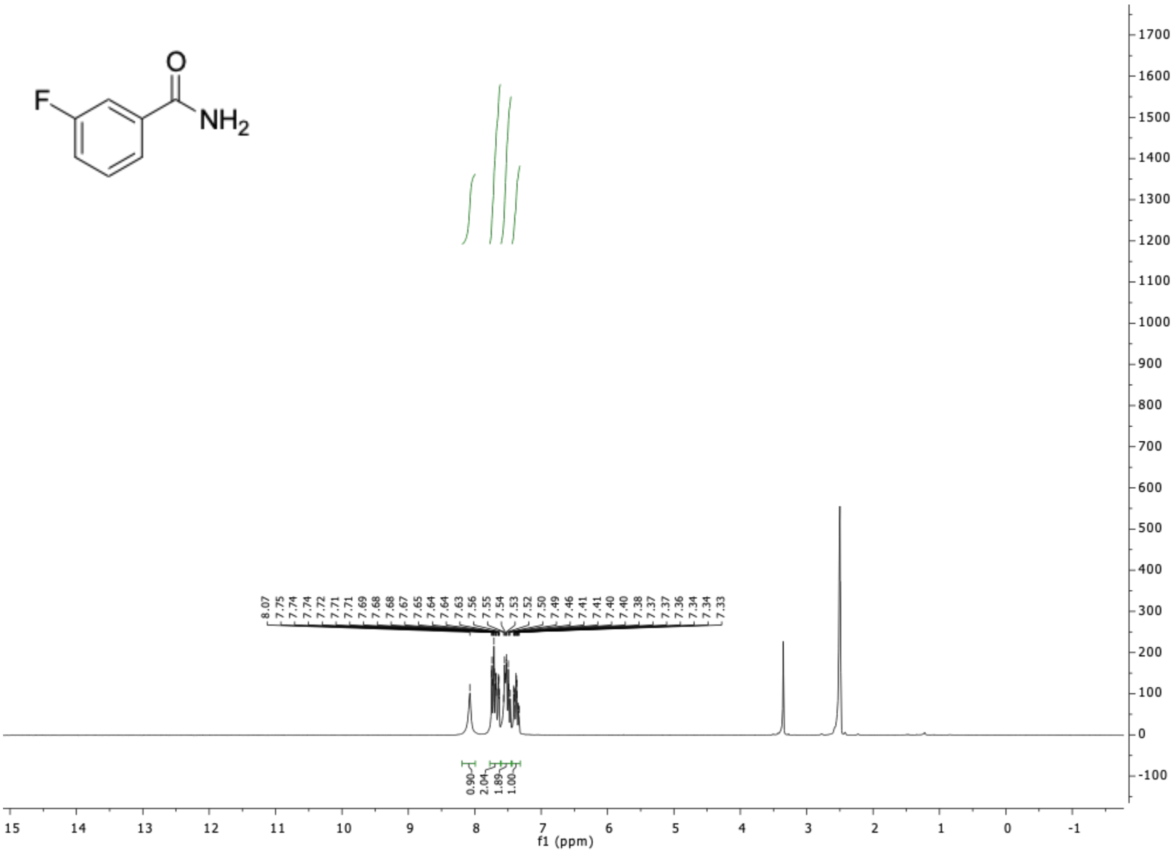

^{13}C{^{1}H} NMR (62.5 MHz, DMSO-d$_6$)

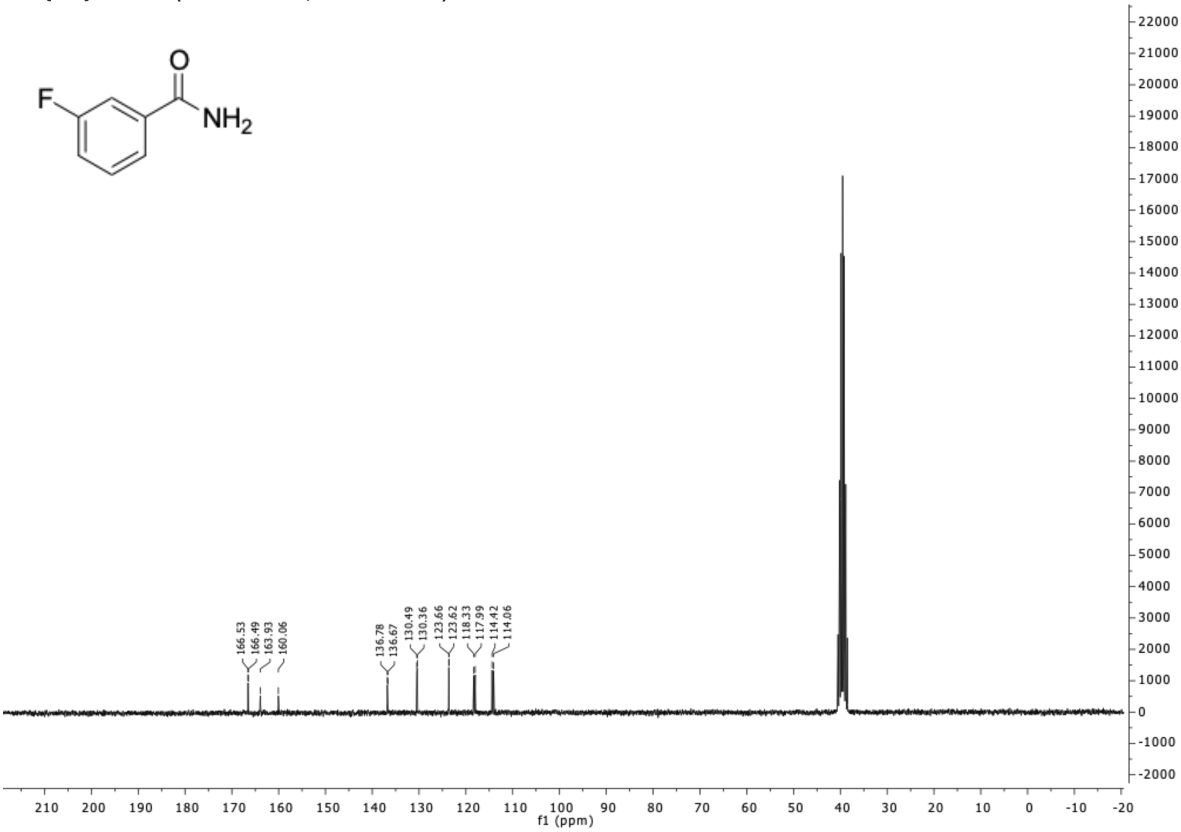

Benzamide (2b)

^{1}H NMR (250 MHz, DMSO-d$_6$)

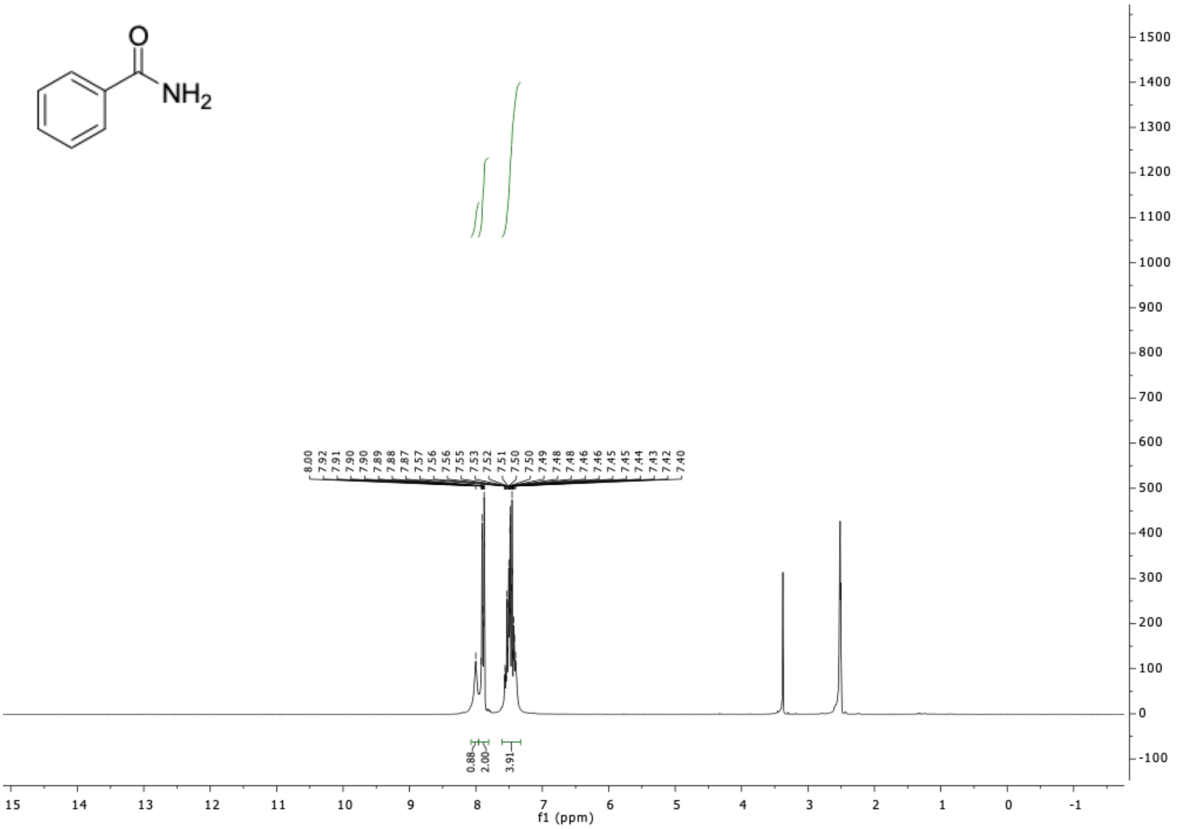

$^{13}C\{^{1}H\}$ NMR (62.5 MHz, DMSO-d$_6$)

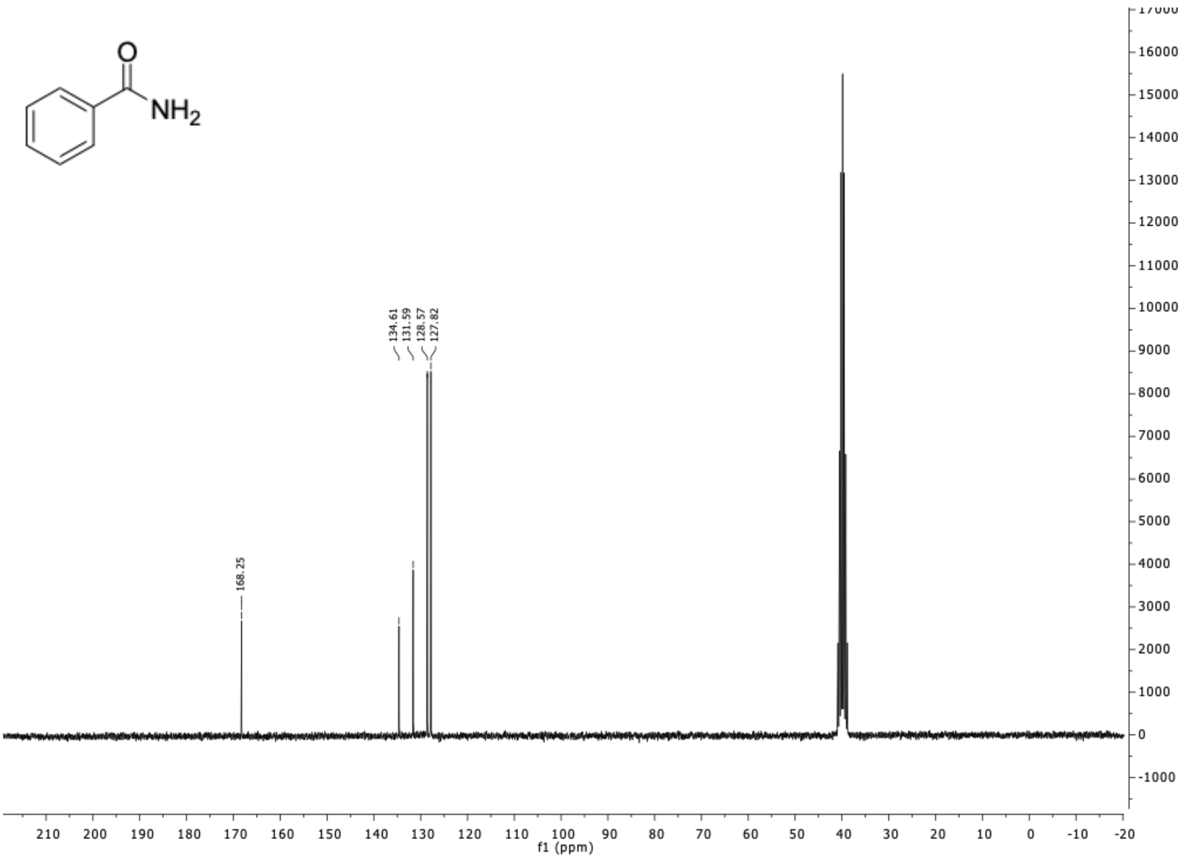

4-Methylbenzamide (2c)

^{1}H NMR (300 MHz, $CDCl_3$)

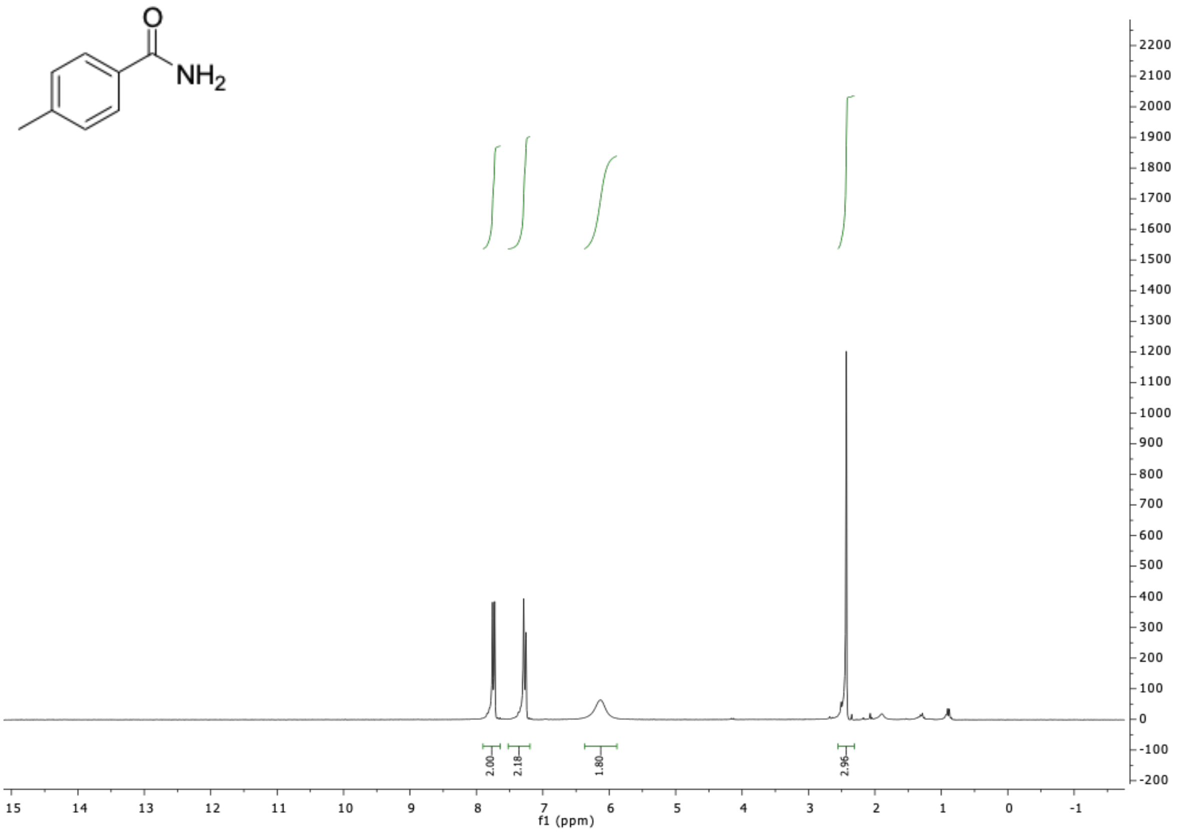

^{13}C{^{1}H} NMR (75 MHz, $CDCl_3$)

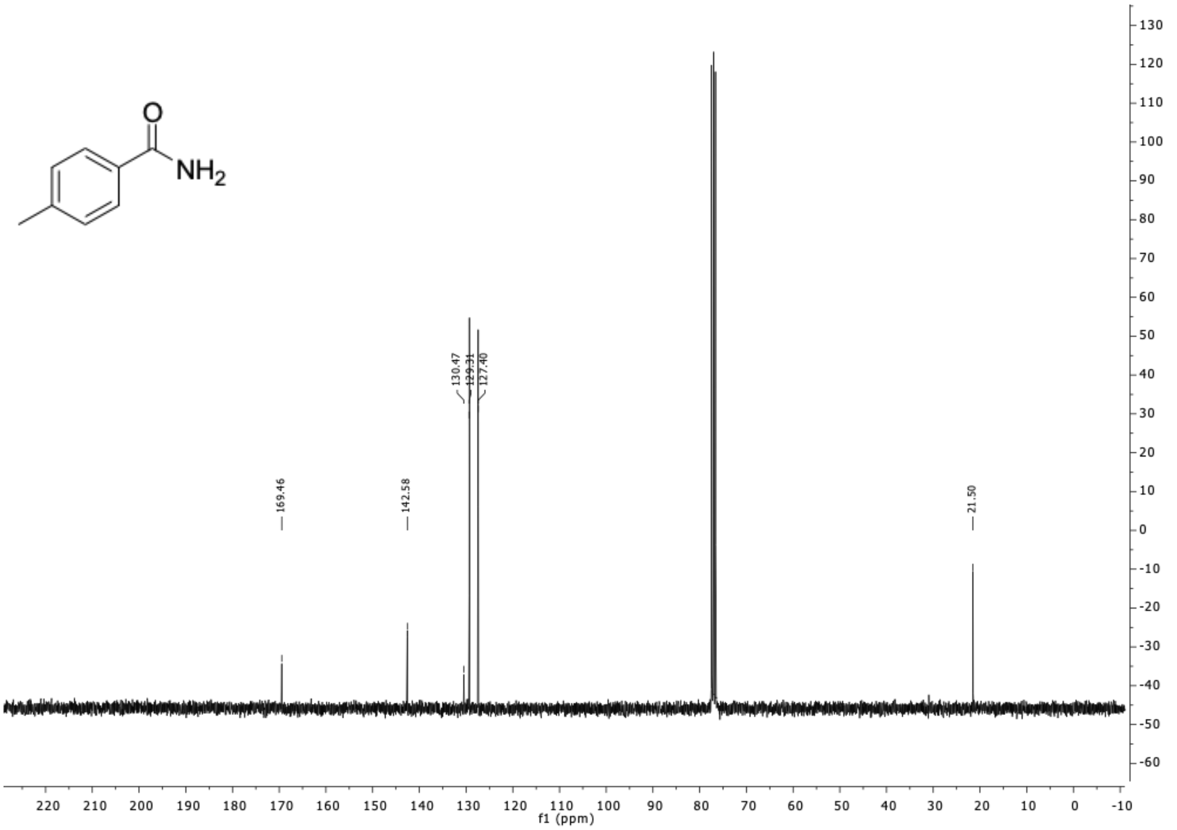

Cinnamide (2d)

^{1}H NMR (250 MHz, DMSO-d6)

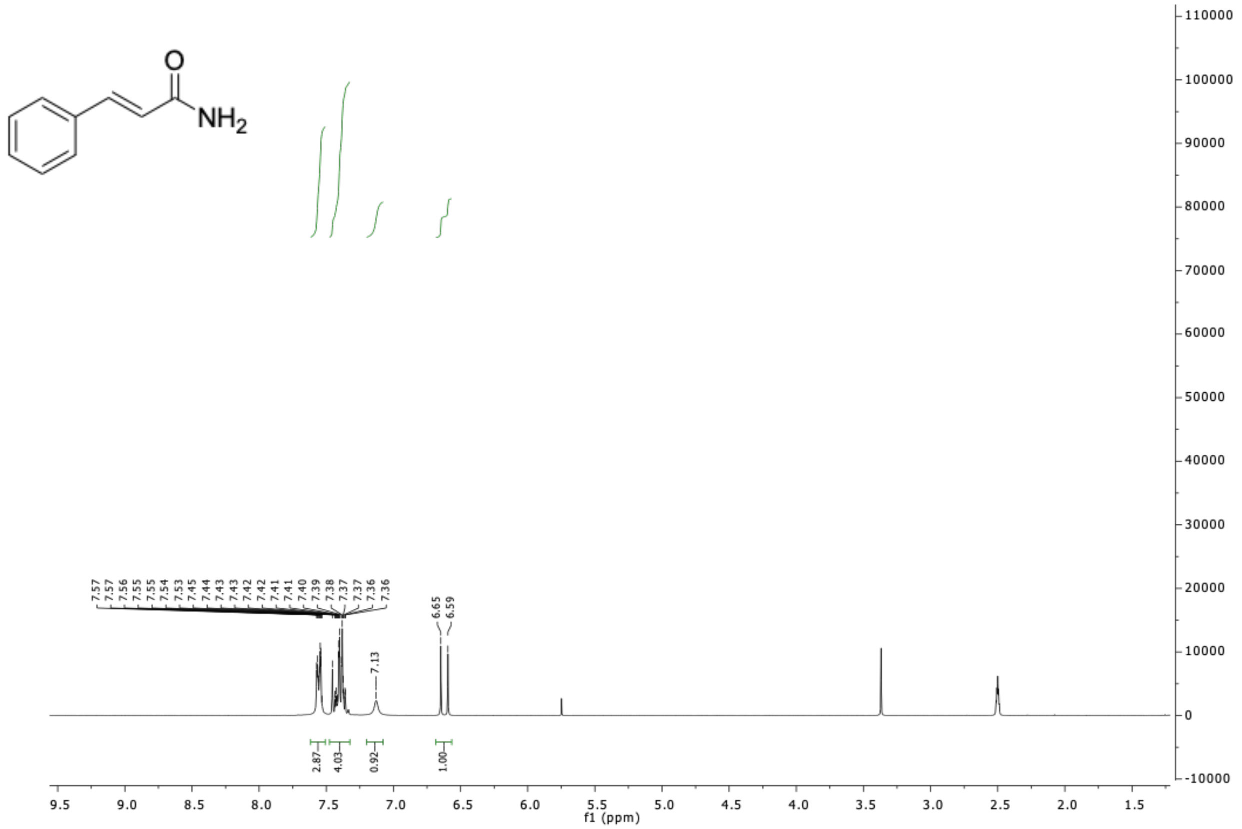

$^{13}C\{^{1}H\}$ NMR (62.5 MHz, DMSO-d6)

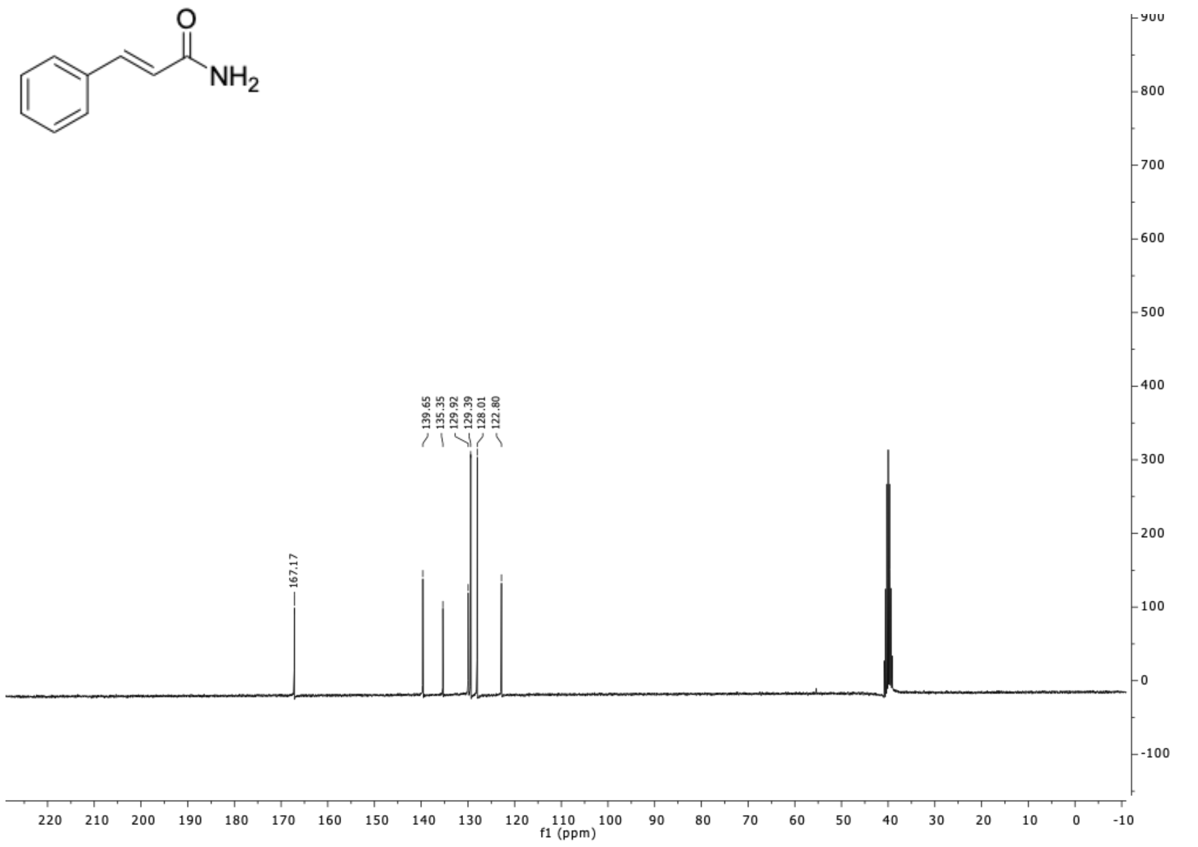

2,2-Diethoxyacetamide (2e)

^{1}H NMR (300 MHz, $CDCl_3$)

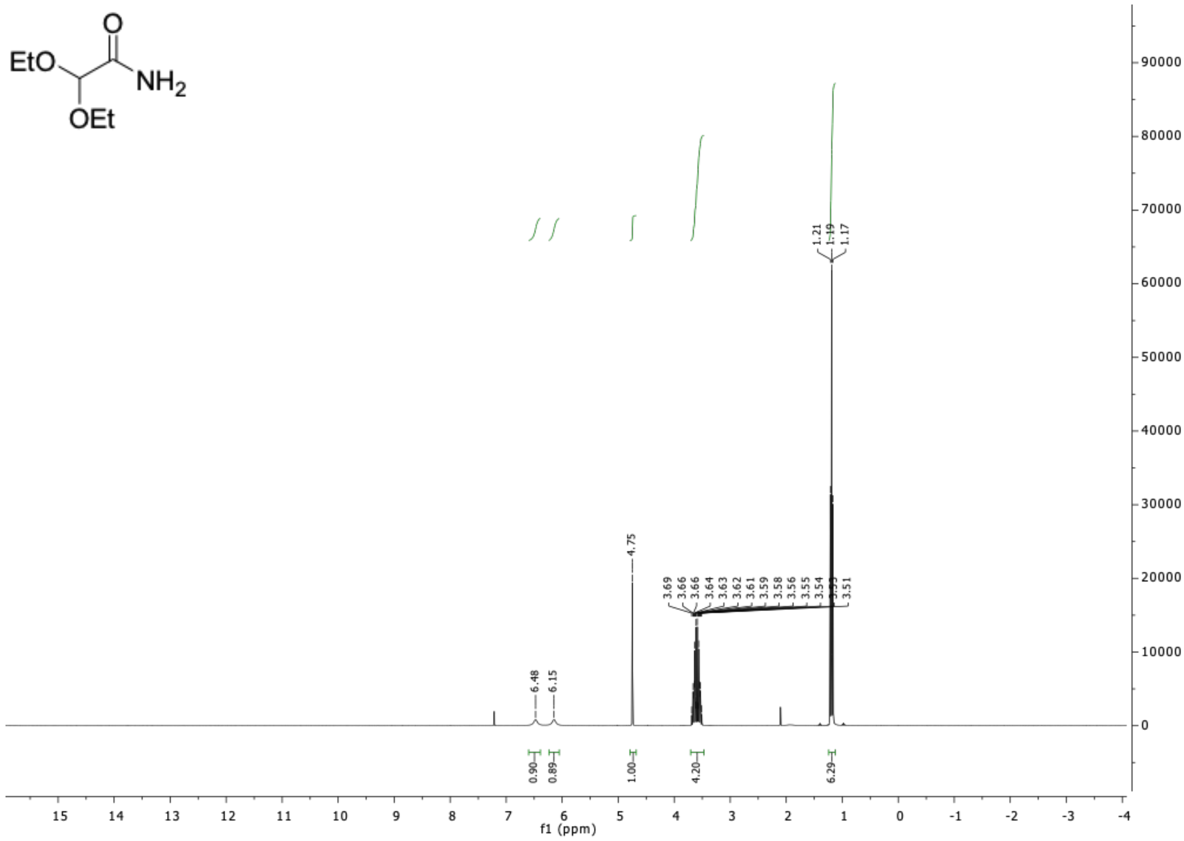

$^{13}C\{^{1}H\}$ NMR (75 MHz, $CDCl_3$)

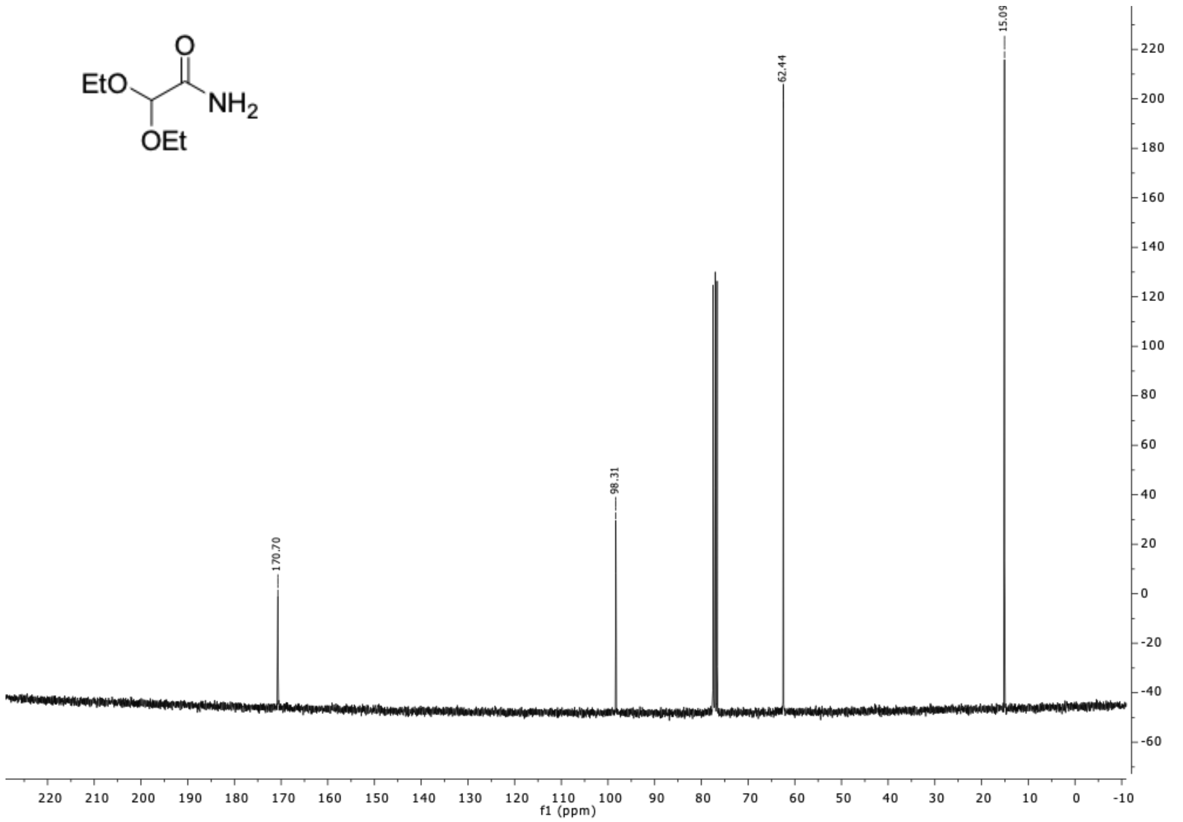

Palmitamide (2f)

^{1}H NMR (300 MHz, $CDCl_3$)

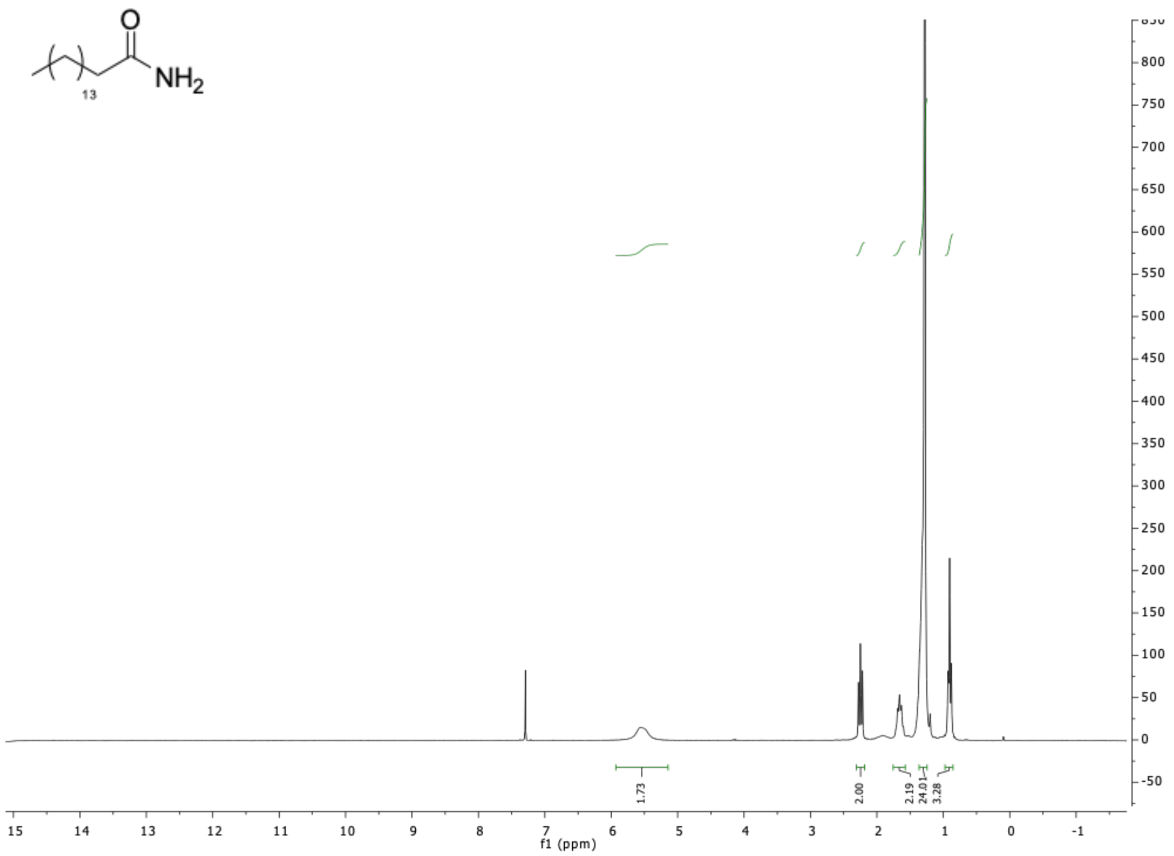

$^{13}C\{^{1}H\}$ NMR (75 MHz, $CDCl_3$)

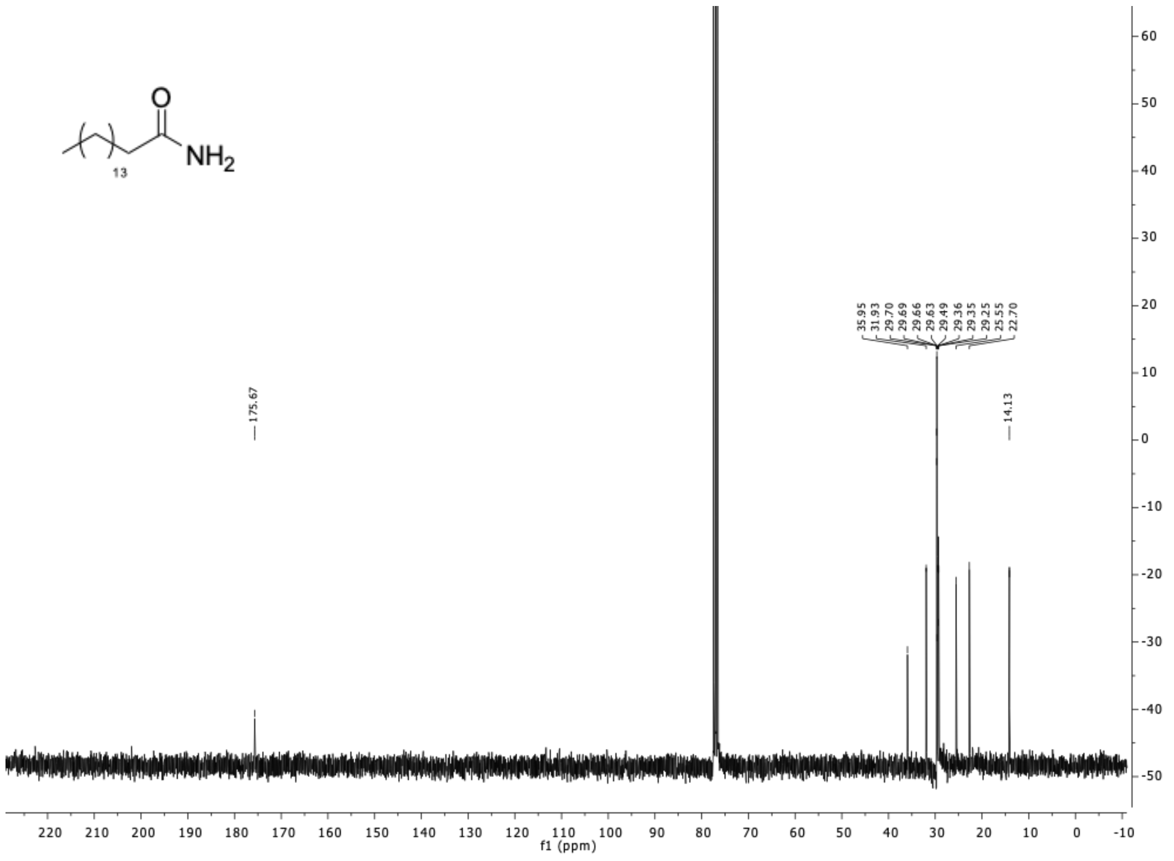

Cyclohexanecarboxamide (2g)

^{1}H NMR (250 MHz, $CDCl_3$)

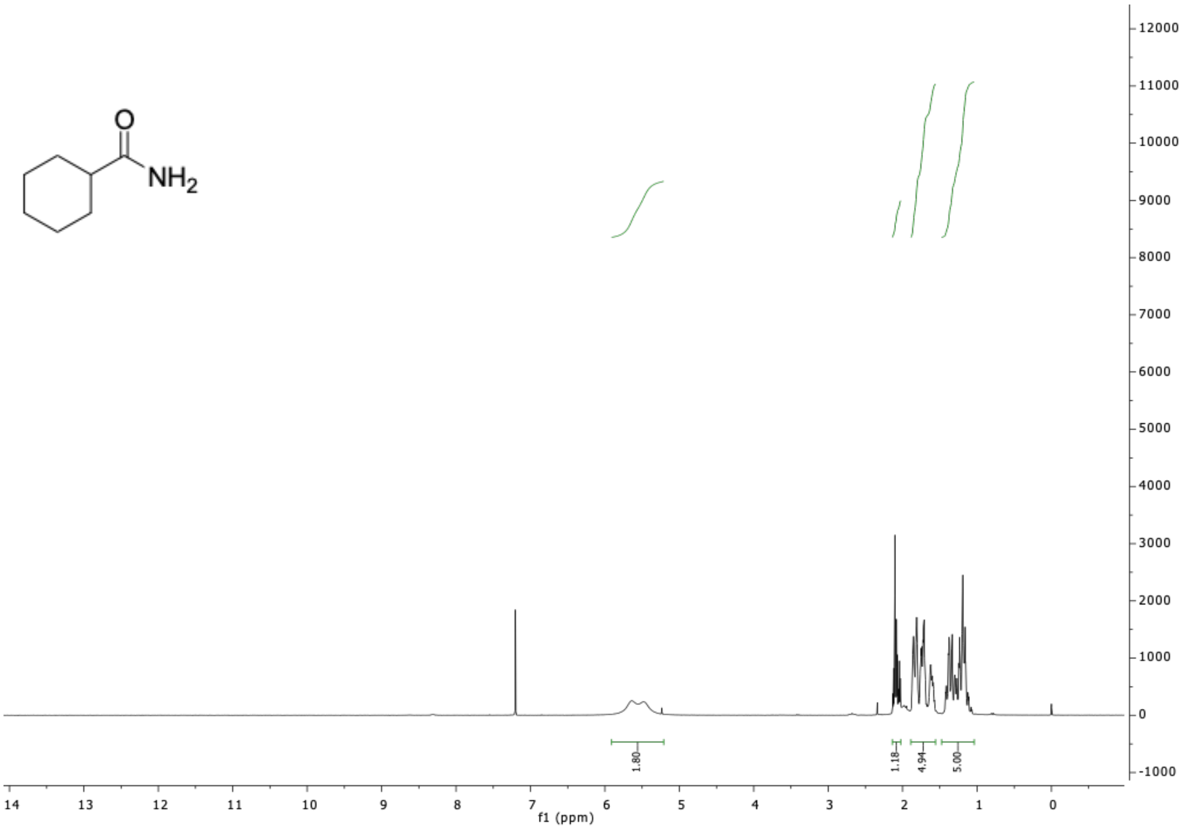

$^{13}C\{^{1}H\}$ NMR (62.5 MHz, $CDCl_3$)

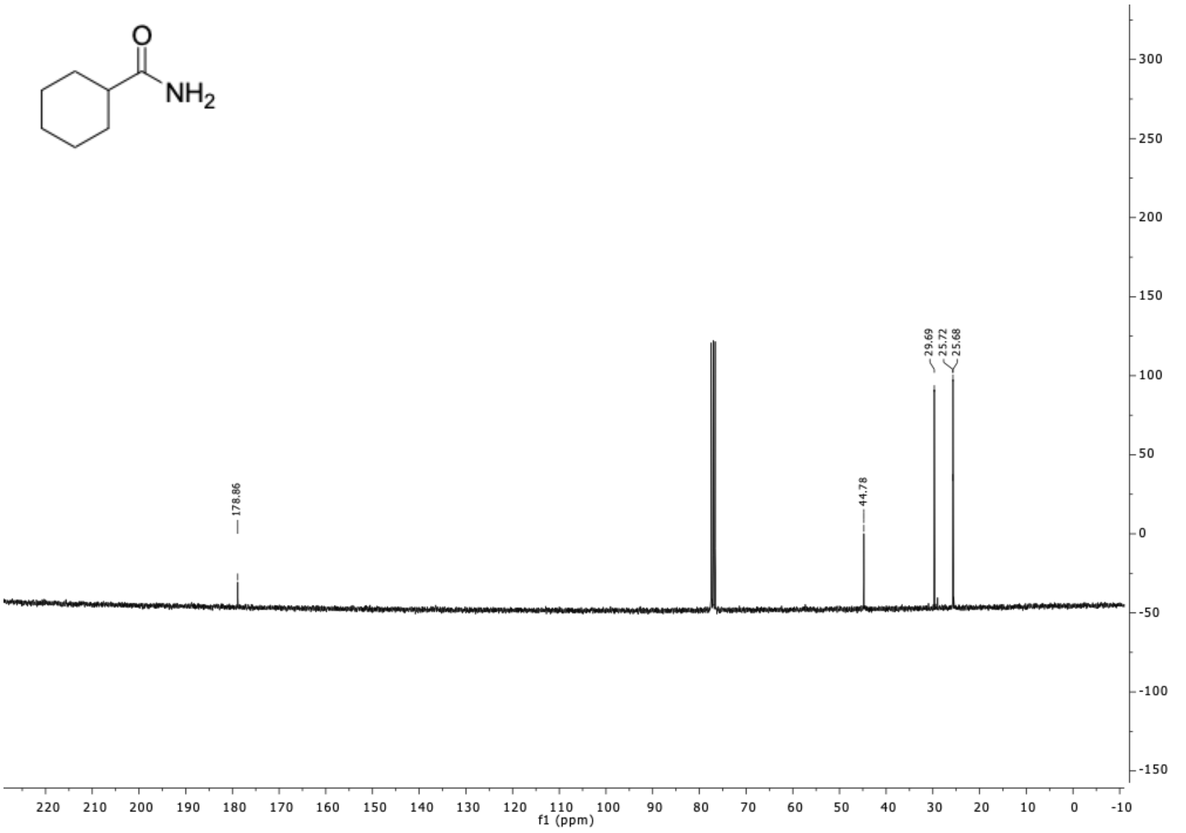

Pyridine 4-carboxamide (isonicotinamide) (2h)

^{1}H NMR (250 MHz, DMSO-d$_6$)

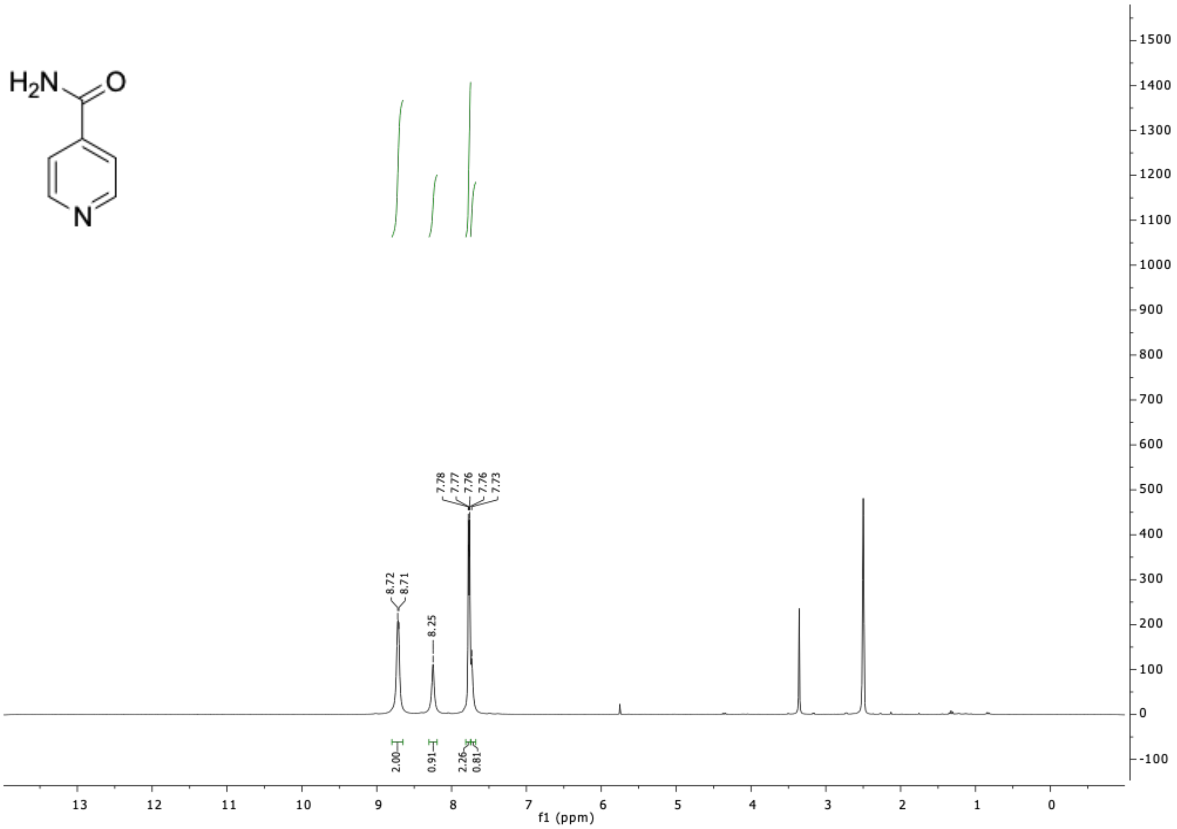

$^{13}C\{^{1}H\}$ NMR (62.5 MHz, DMSO-d$_6$)

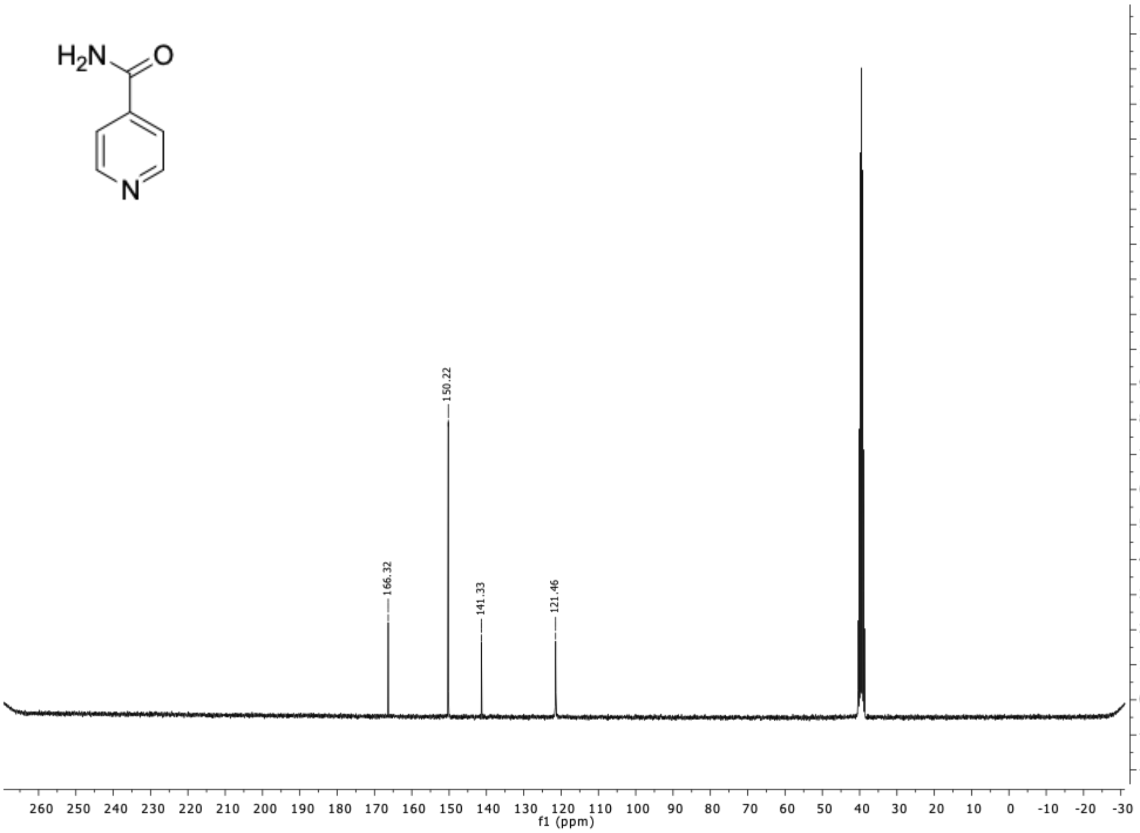

Pyridine-3-carboxamide (nicotinamide, niacinamide) (2i)

^{1}H NMR (250 MHz, DMSO-d$_6$)

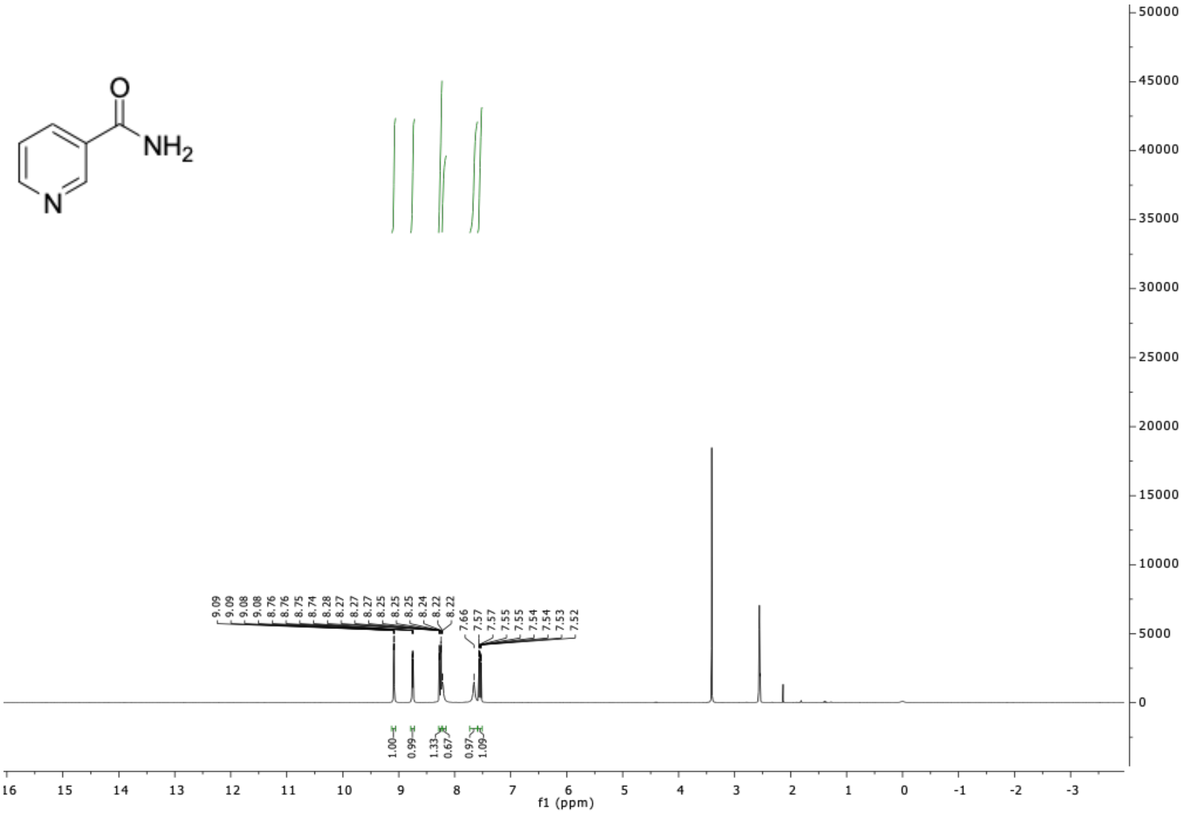

^{13}C{^{1}H} NMR (62.5 MHz, DMSO-d$_6$)

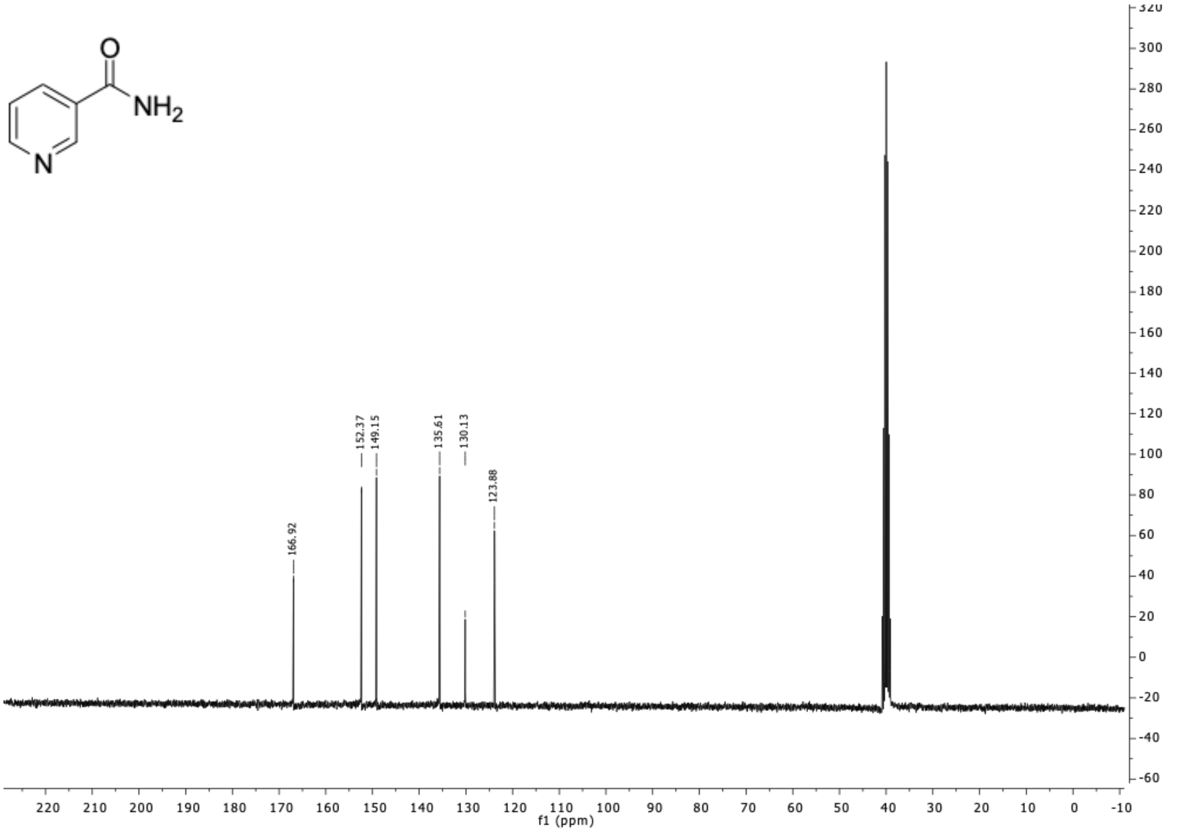

Thiophene-2-carboxamide (2j)

^{1}H NMR (250 MHz, DMSO-d$_6$)

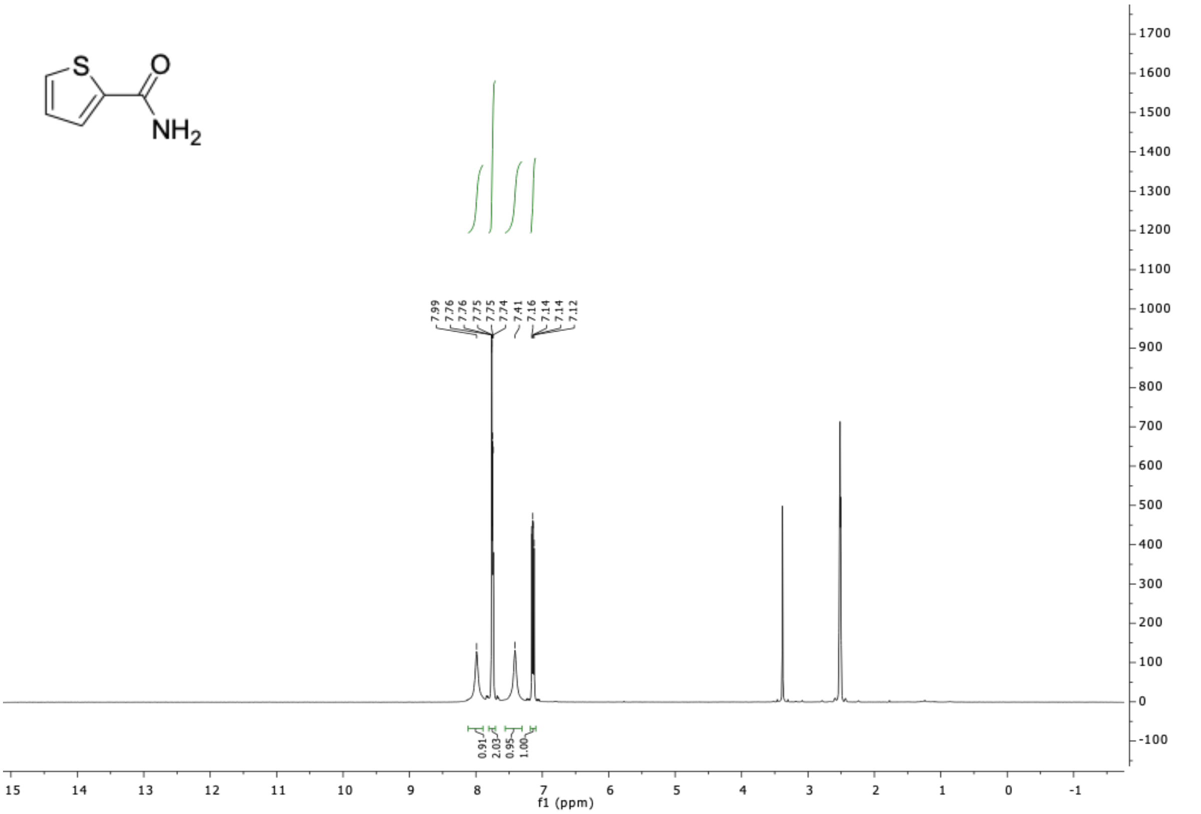

^{13}C{^{1}H} NMR (62.5 MHz, DMSO-d$_6$)

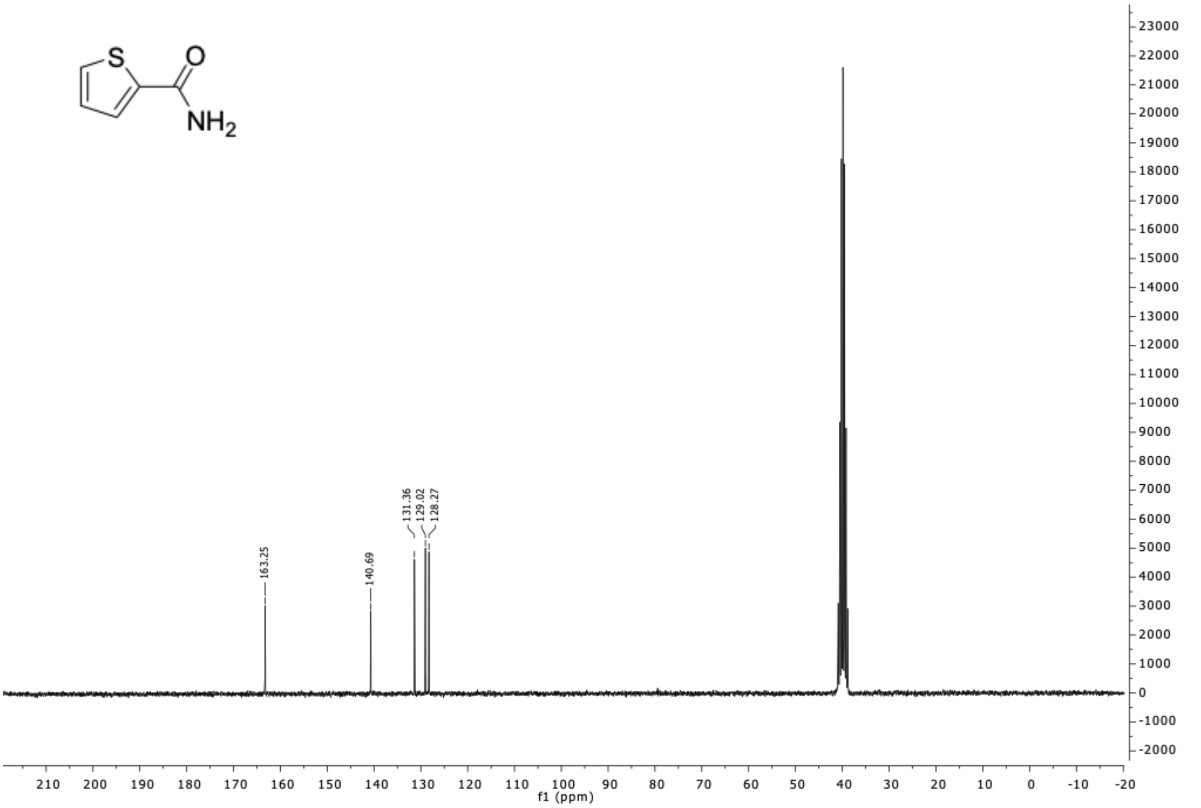

2-Phenylacetamide (2k)

^{1}H NMR (250 MHz, $CDCl_3$)

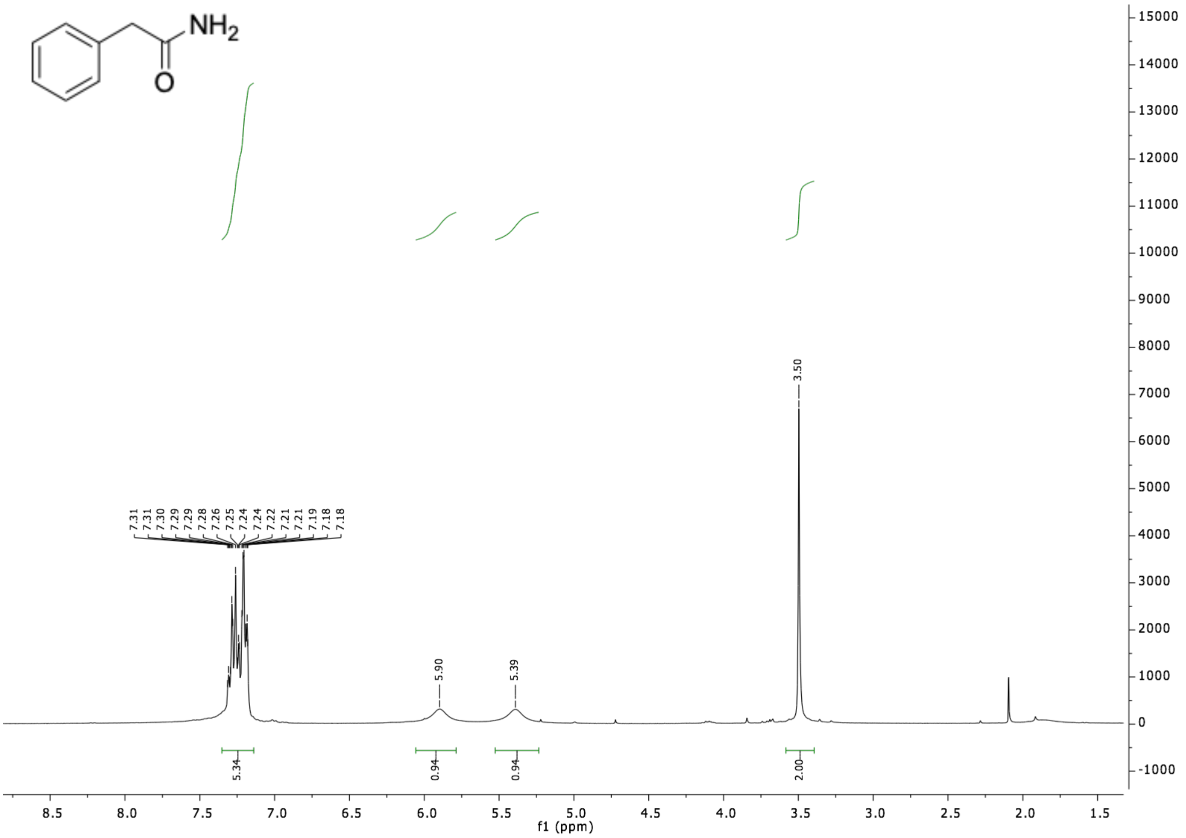

$^{13}C\{^{1}H\}$ NMR (62.5 MHz, $CDCl_3$)

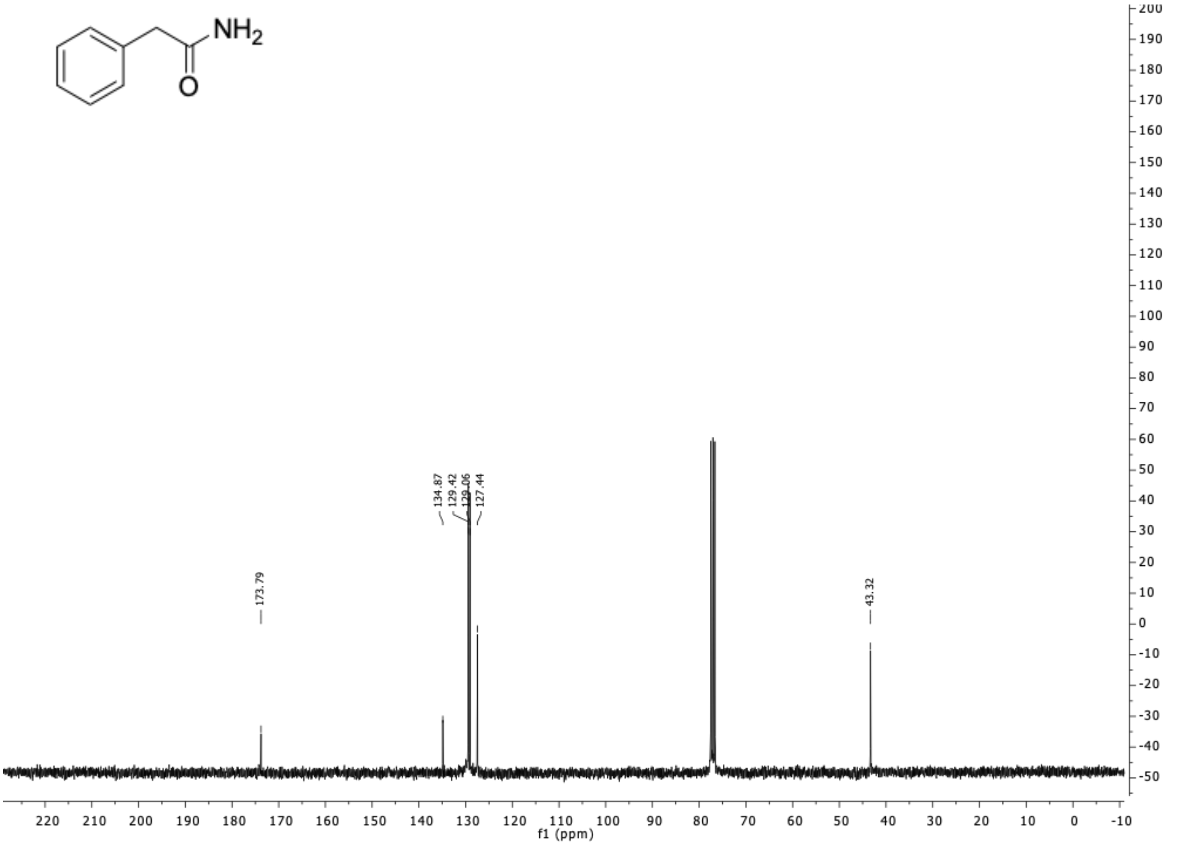

2-(Piperidin-1-yl)acetamide (2l)

^{1}H NMR (250 MHz, $CDCl_3$)

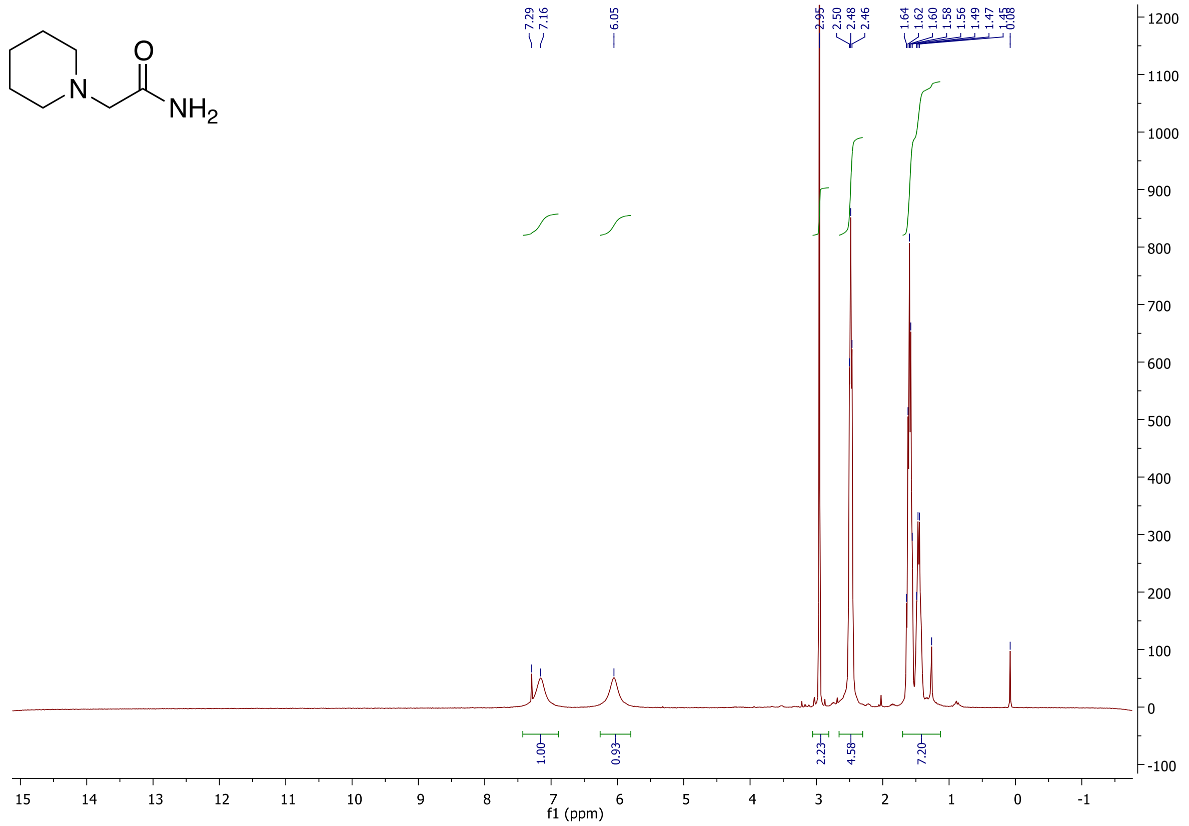

$^{13}C\{^{1}H\}$ NMR (62.5 MHz, $CDCl_3$)

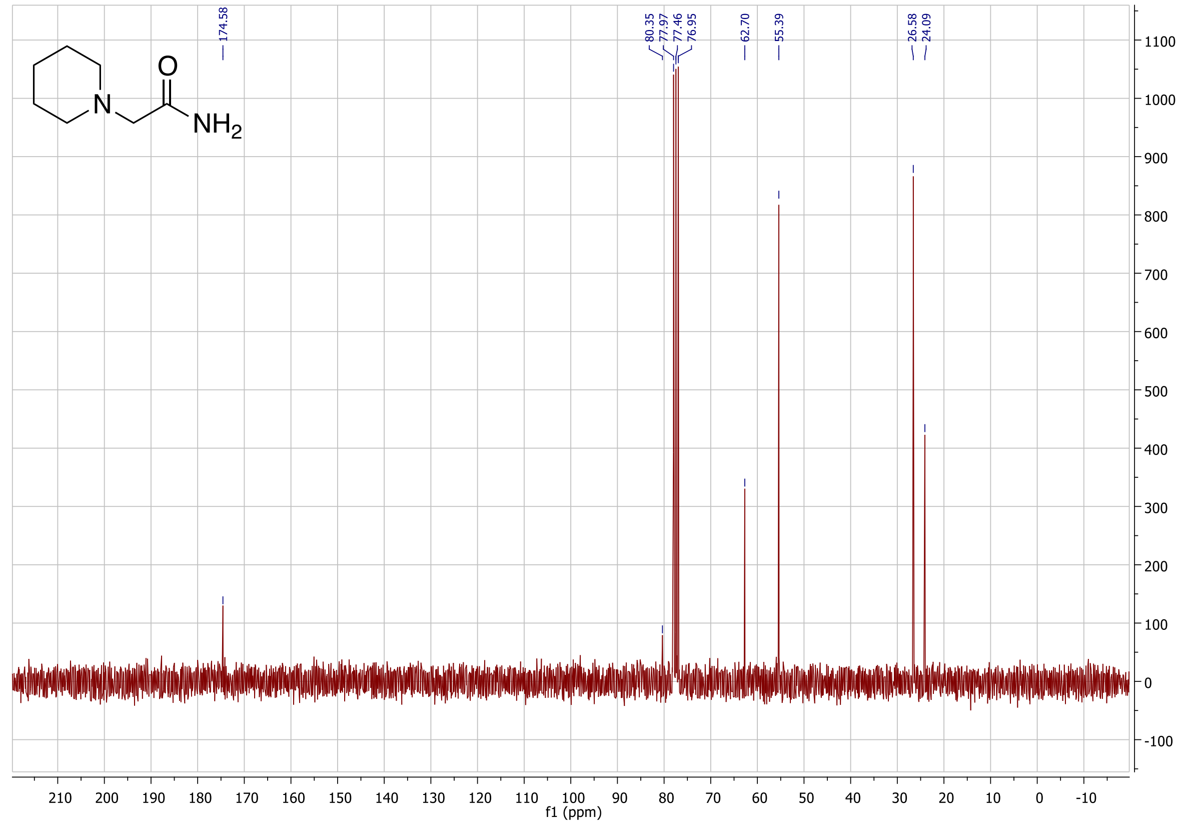

(+)-Phenylalaninamide (2m)

^{1}H NMR (300 MHz, CD_3OD)

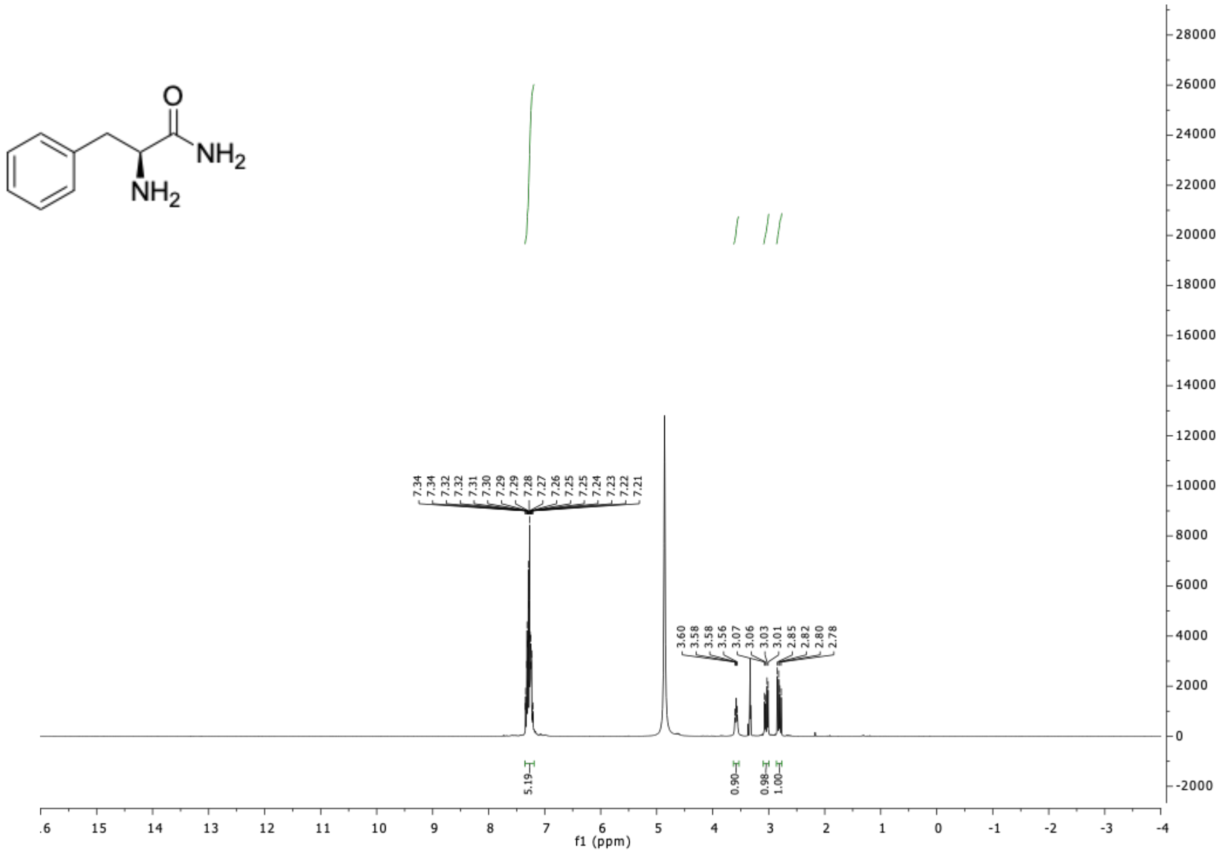

$^{13}C\{^1H\}$ NMR (75 MHz, CD_3OD)

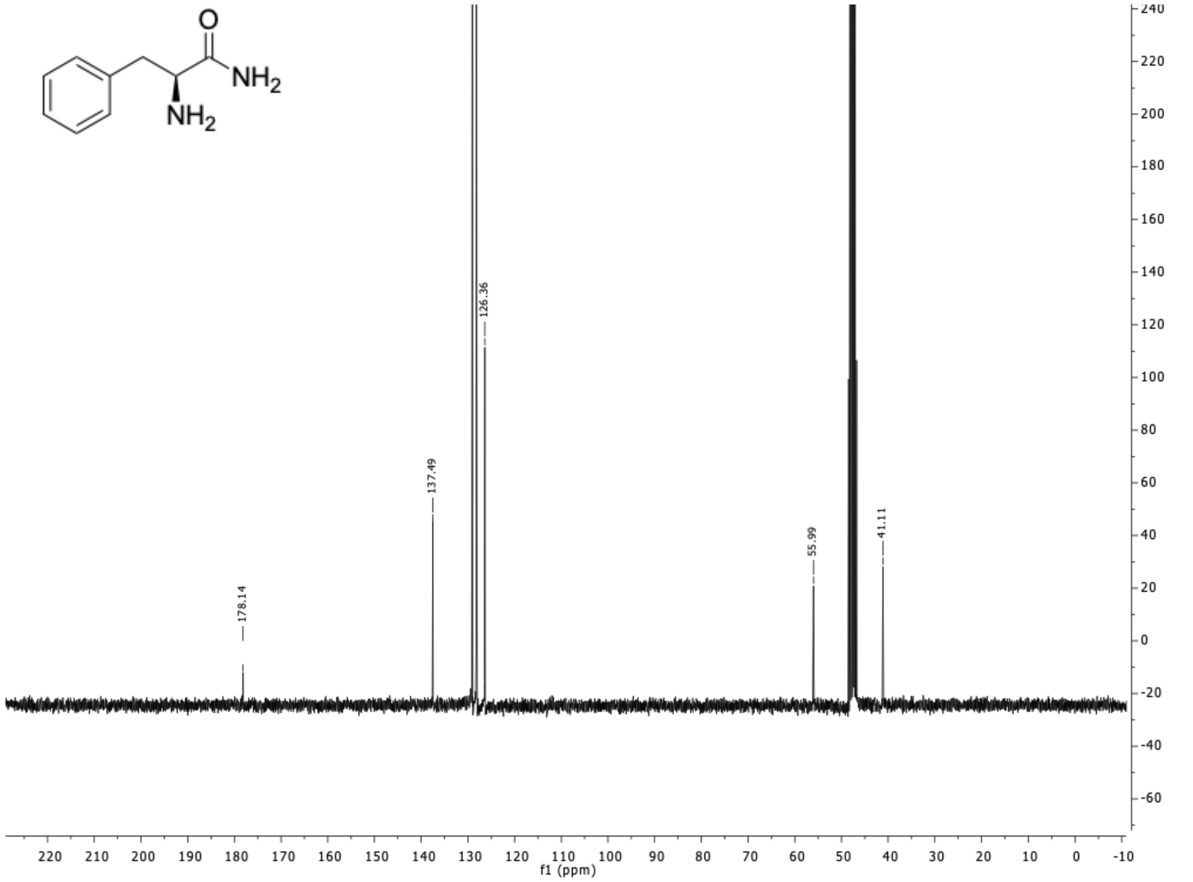

(+)-Methioninamide (2n)

^{1}H NMR (300 MHz, CD_3OD)

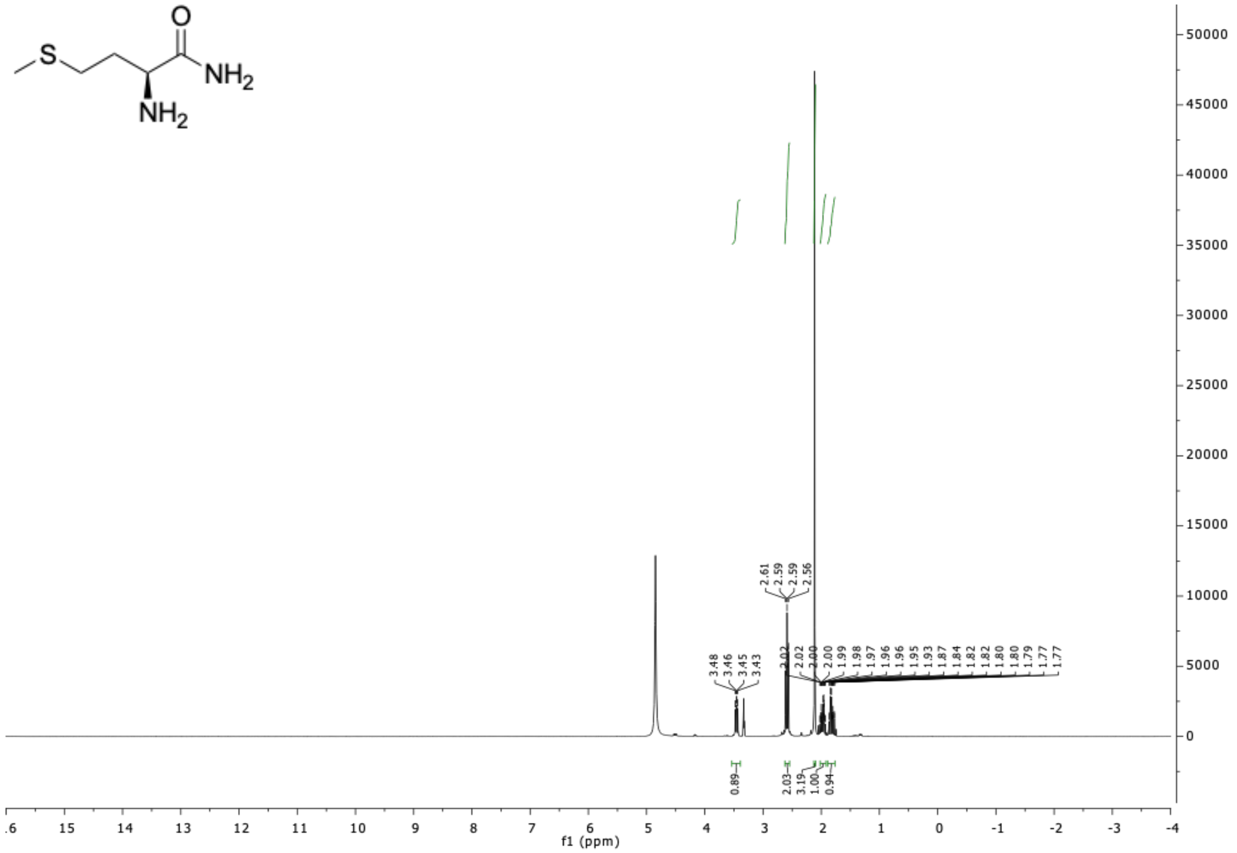

$^{13}C\{^{1}H\}$ NMR (75 MHz, CD_3OD)

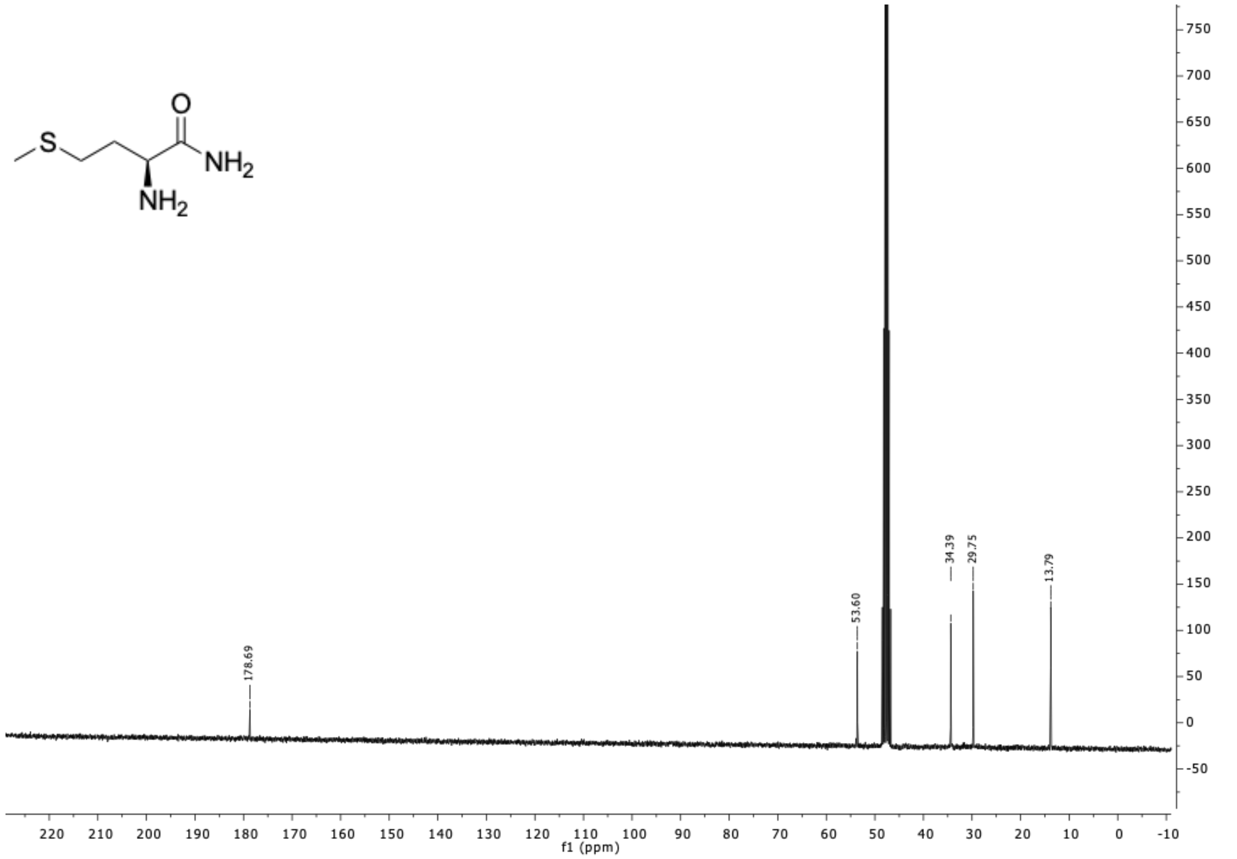

(S)-*tert*-Butyl (1-((2-amino-2-oxoethyl)amino)-3-(1H-indol-3-yl)-1-oxopropan-2-yl)carbamate (2o)

^{1}H NMR (250 MHz, $CDCl_3$)

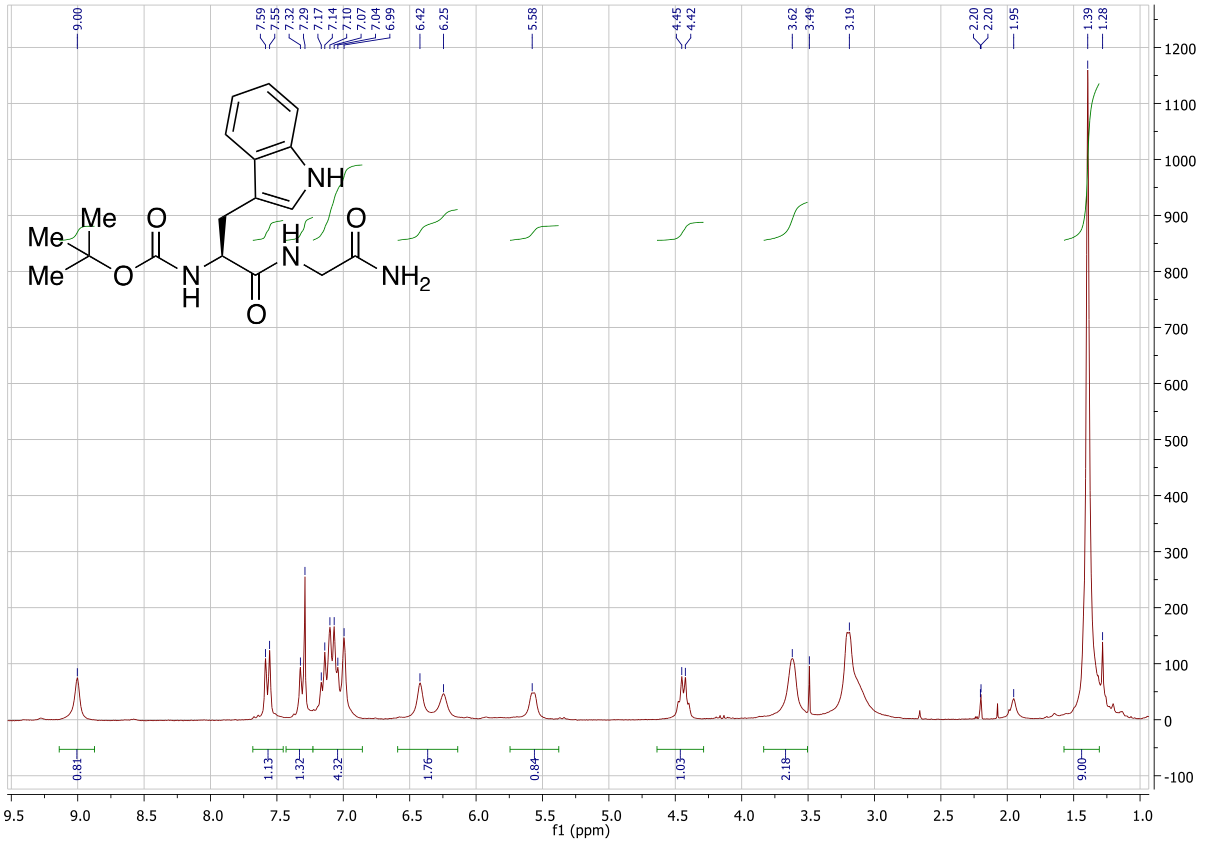

$^{13}C\{^{1}H\}$ NMR (62.5 MHz, $CDCl_3$)

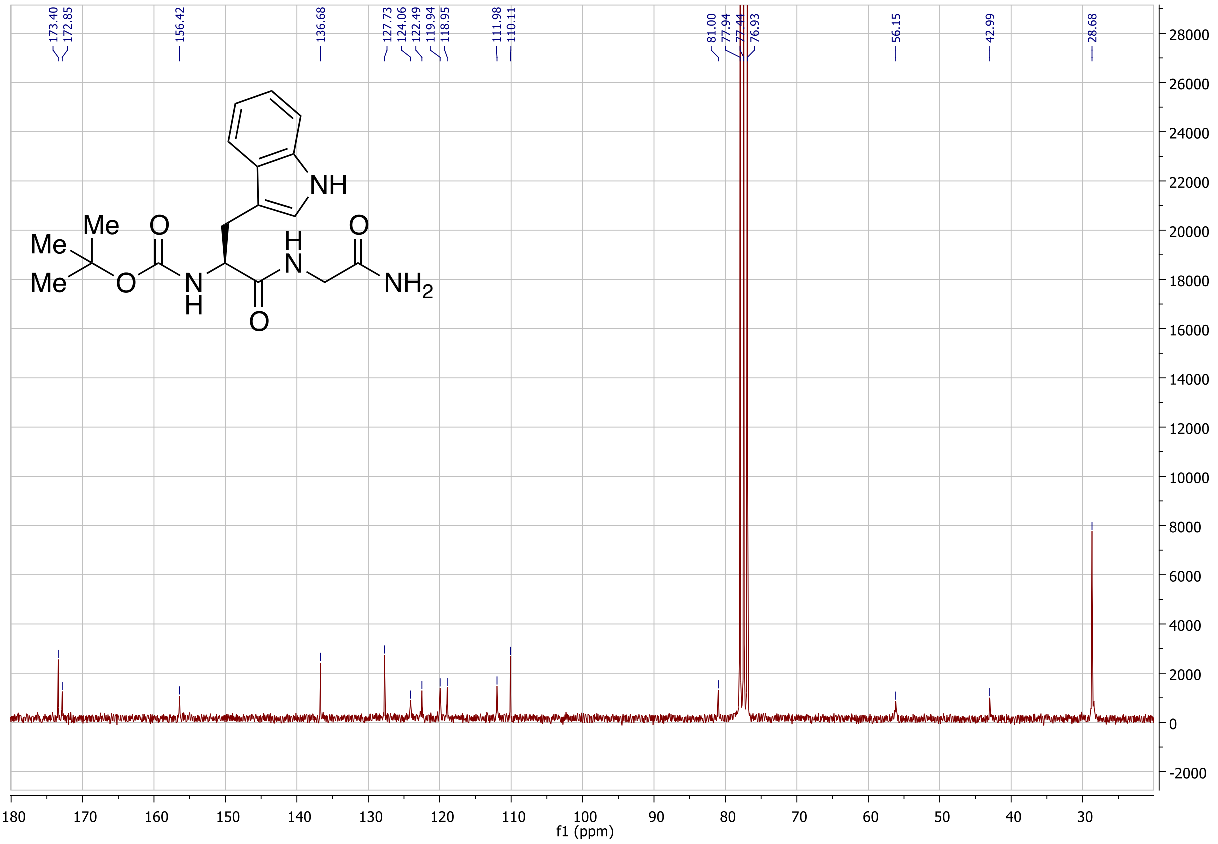

(*S*)-*tert*-Butyl (1-((2-amino-2-oxoethyl)amino)-1-oxo-3-phenylpropan-2-yl)carbamate (2p)

^{1}H NMR (250 MHz, $CDCl_3$)

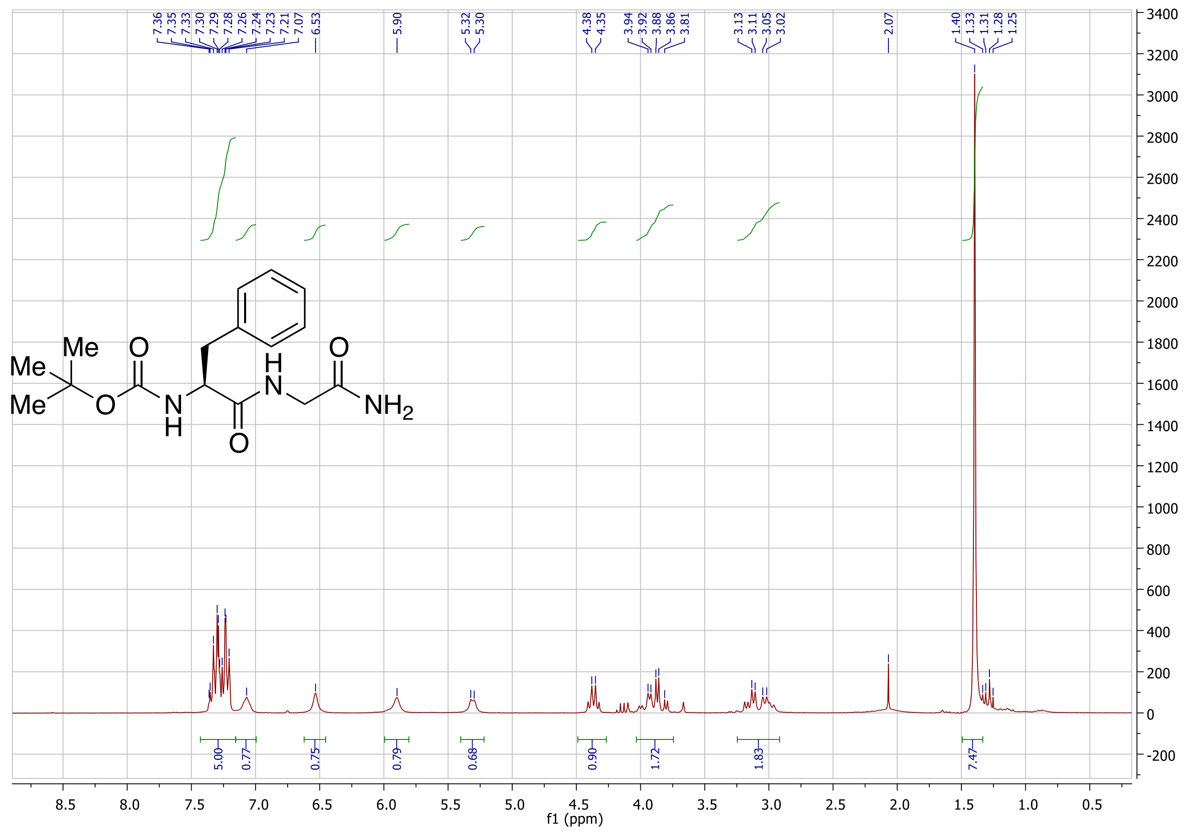

^{13}C{^{1}H} NMR (62.5 MHz, $CDCl_3$)

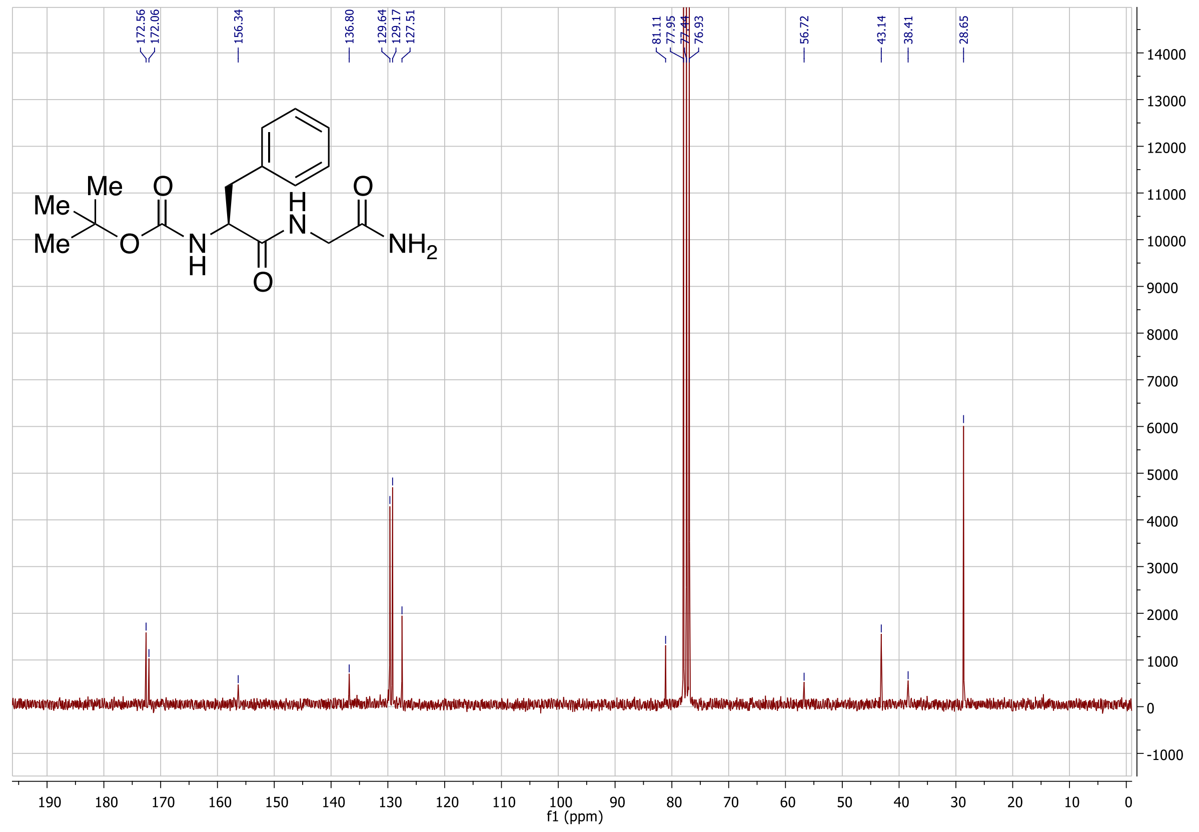

(*S*)-*tert*-Butyl (1-((2-amino-2-oxoethyl)amino)-1-oxopropan-2-yl)carbamate (2q)

^{1}H NMR (250 MHz, $CDCl_3$)

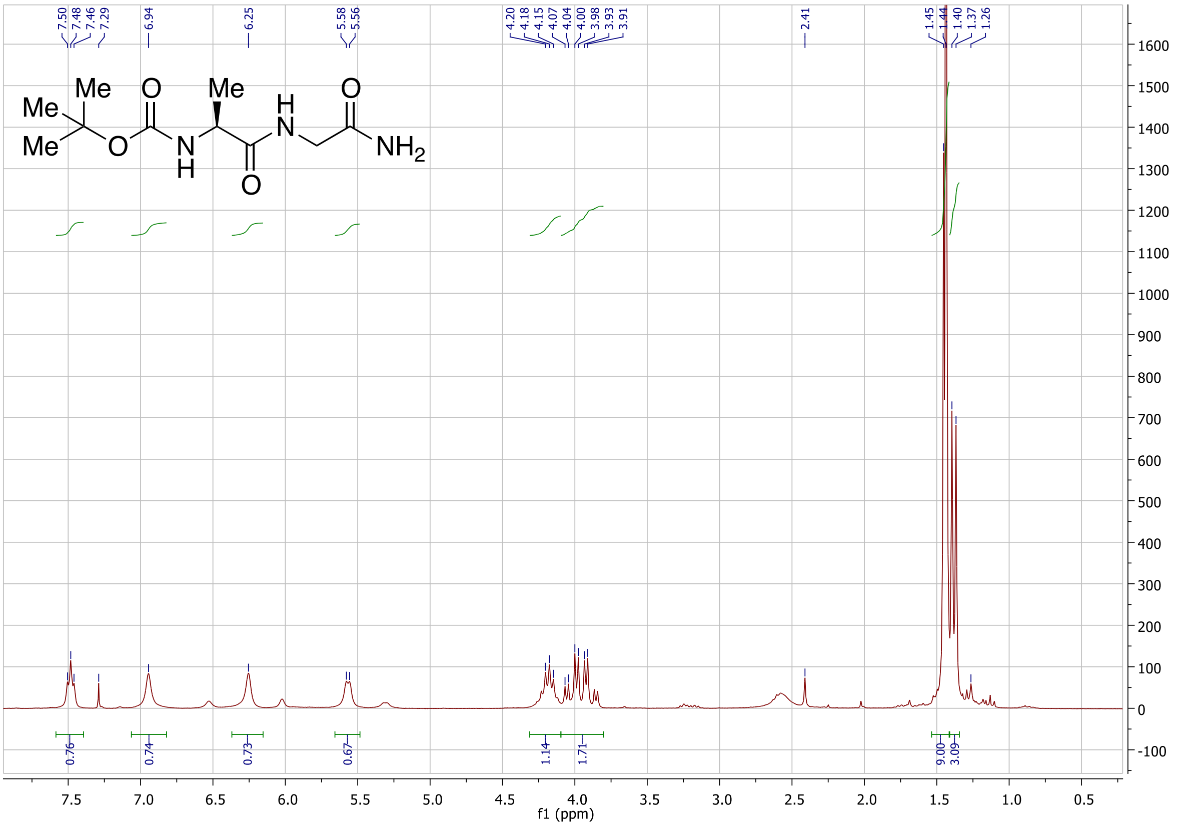

$^{13}C\{^{1}H\}$ NMR (62.5 MHz, $CDCl_3$)

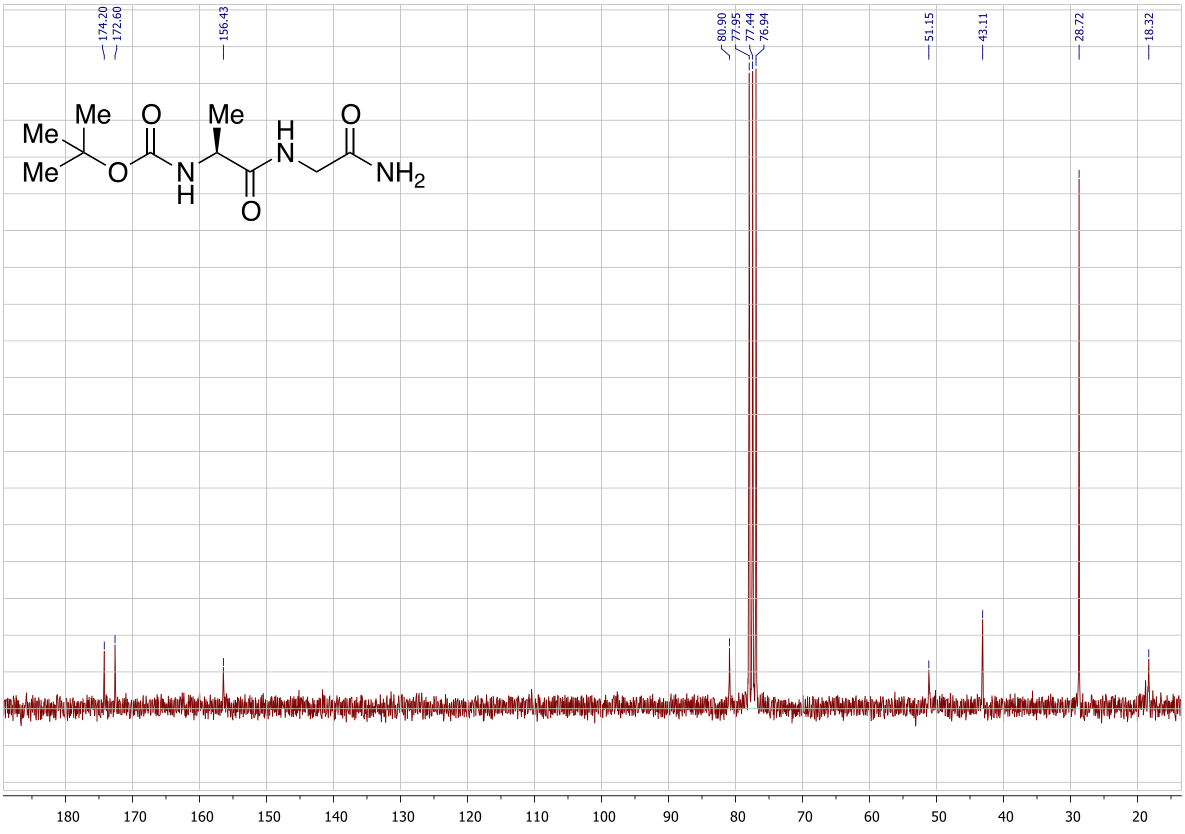

3-(2-Hydroxyphenyl)propanamide (2r)

^{1}H NMR (250 MHz, DMSO-d_6)

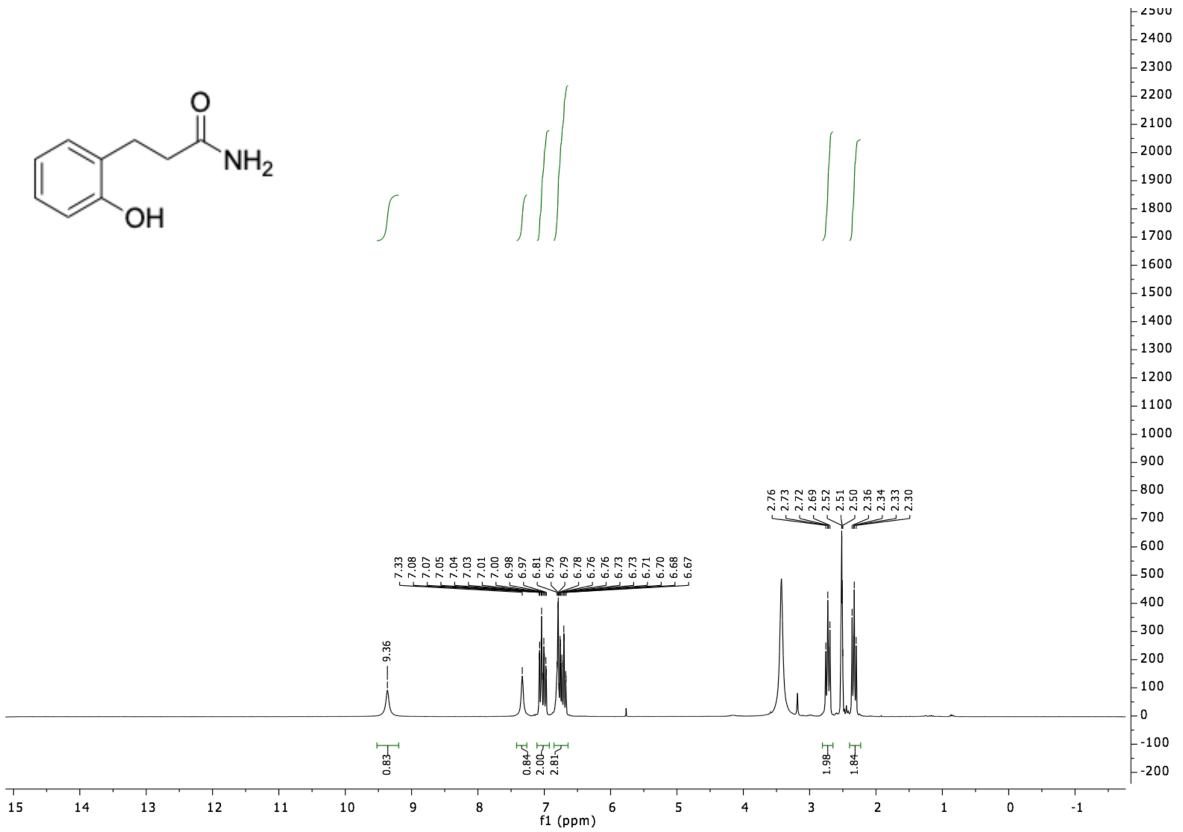

$^{13}C\{^{1}H\}$ NMR (62.5 MHz, DMSO-d_6)

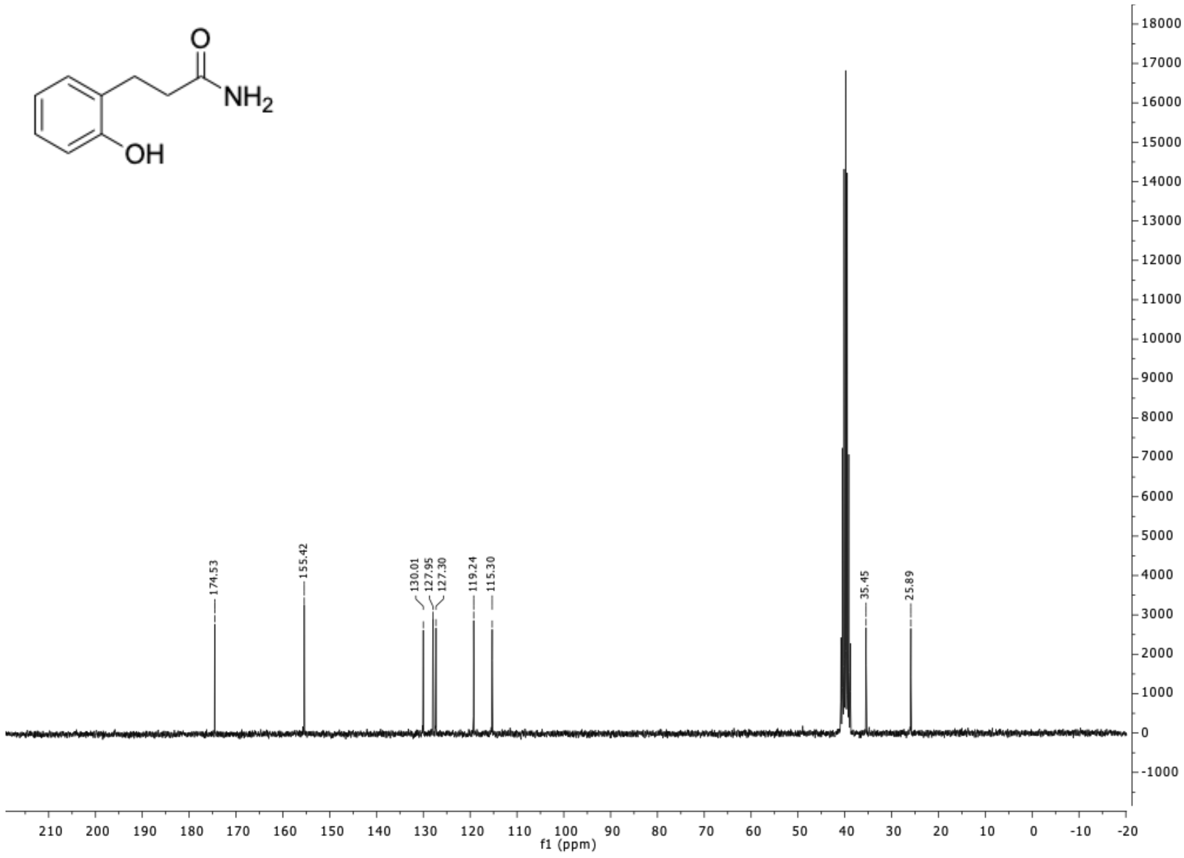

1-(2,6-Difluorobenzyl)-1*H*-1,2,3-triazole-4-carboxamide (rufinamide) (6)

^{1}H NMR (250 MHz, DMSO-d$_6$)

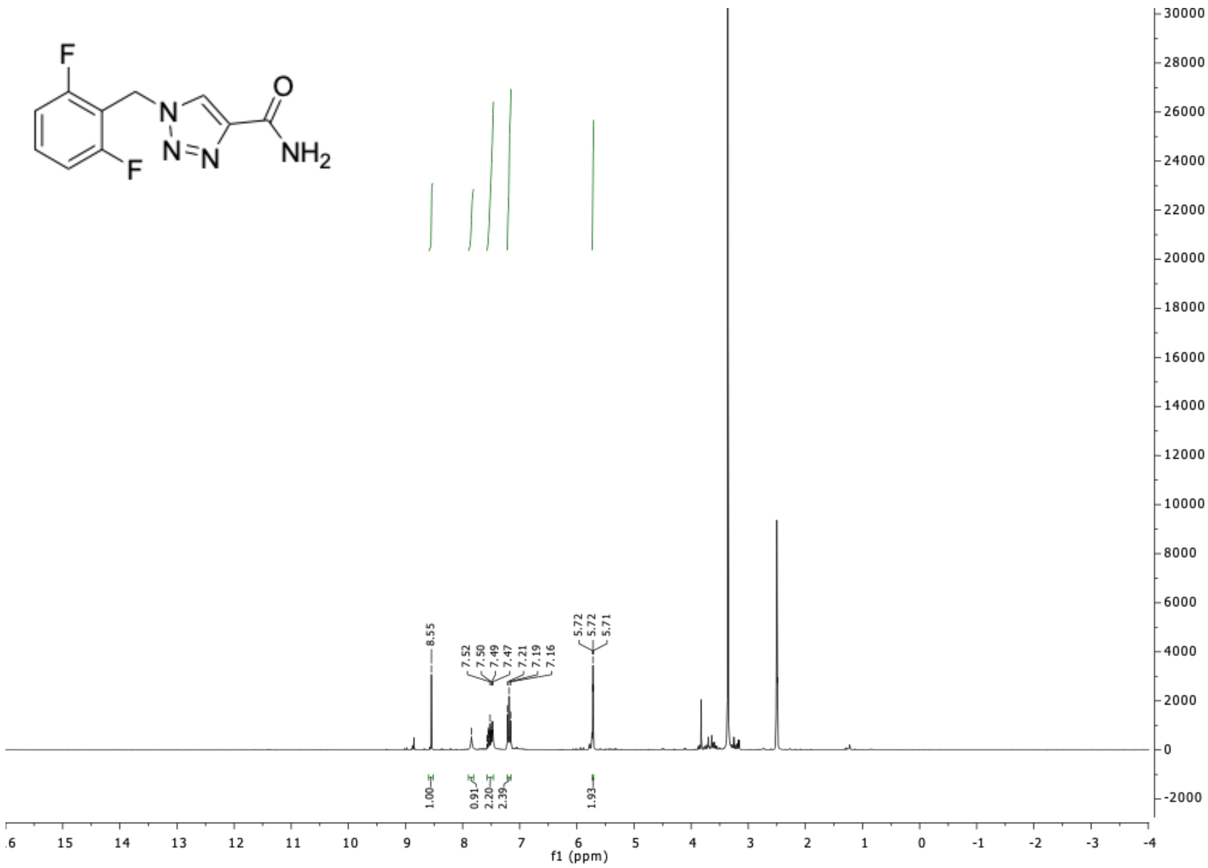

^{13}C{^{1}H} NMR (62.5 MHz, DMSO-*d*$_6$)

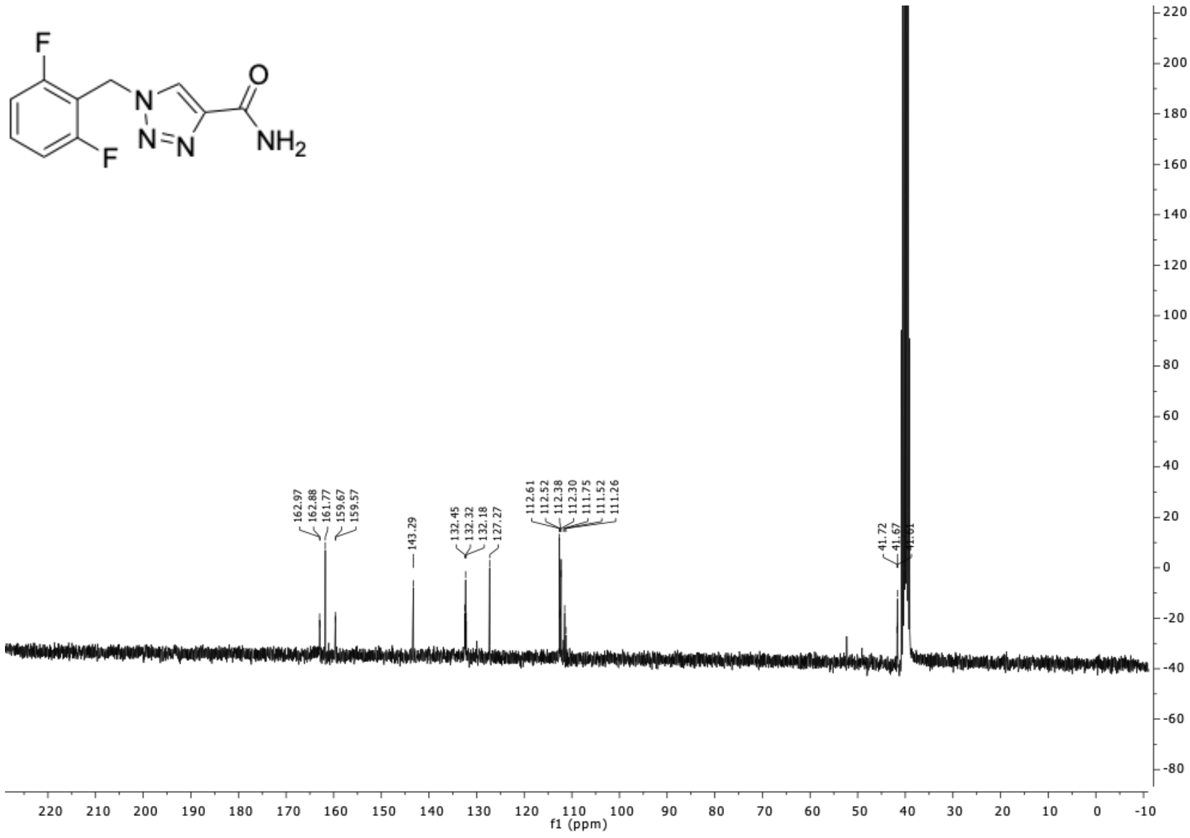

Supplement: Supplementary file 1 — jo1c02350_si_001.pdf [file jo1c02350_si_001.pdf]
